# Meta-analysis-derived estimates of stressor–response associations for riverine organism groups

**DOI:** 10.1038/s41559-025-02884-4

**Published:** 2025-11-11

**Authors:** Willem Kaijser, Michelle Musiol, Andrea R. Schneider, Sebastian Prati, Verena S. Brauer, Rike Bayer, Sebastian Birk, Mario Brauns, Louisa Dunne, Julian Enss, Luan Farias, Christian K. Feld, Lena Feldhaus, Svenja M. Gillmann, Kamil Hupało, Stephen E. Osakpolor, Sarah L. M. Olberg, Iris Madge Pimentel, Ralf B. Schäfer, Christian Schlautmann, Jessica Schwelm, Bernd Sures, Cornelia S. Wagner, Nicole E. Wells, Franziska Wenskus, Christian Schürings, Daniel Hering

**Affiliations:** 1 Aquatic Ecology, University of Duisburg-Essen, Essen, Germany; 2 Centre for Water and Environmental Research (ZWU), Essen, Germany; 3 Research Center One Health Ruhr, University Alliance Ruhr, University of Duisburg-Essen, Essen, Germany; 4 Environmental Microbiology and Biotechnology, University of Duisburg-Essen, Essen, Germany; 5 Department of River Ecology, Helmholtz Centre for Environmental Research—UFZ, Magdeburg, Germany; 6 Aquatic Ecosystem Research, University of Duisburg-Essen, Essen, Germany; 7 Institute for Environmental Sciences, RPTU University of Kaiserslautern-Landau, Landau, Germany; 8 Ecotoxicology, University of Duisburg-Essen, Essen, Germany; 9 Water Research Group, Unit for Environmental Sciences and Management, North-West University, Potchefstroom, South Africa

**Keywords:** Freshwater ecology, Biodiversity

## Abstract

Freshwater ecosystems, particularly rivers, are experiencing the most rapid biodiversity declines of any biome, driven by several interacting stressors operating across local to global scales. Despite growing research on these interactions, the lack of systematic quantification of individual stressor gradients limits our ability to disentangle their cumulative effects. Here we present a global synthesis of stressor–response relationships across five key riverine organism groups—prokaryotes, algae, macrophytes, invertebrates and fish. We screened 22,120 papers and extracted 276 studies with 1,332 stressor–response relationships. We used generalized linear mixed models and Bayesian meta-analyses to quantify the response to the seven most prevalent stressors. Consistently across taxa, biodiversity loss (taxon richness and evenness) reflected elevated salinity, oxygen depletion and fine sediment accumulation, while the association with nutrient enrichment and warming varied among groups. Predictive tools, including hypothetical outcome plots and partial dependence plots, revealed the interplay of stressors and predicted biodiversity response to stress increase. Our findings establish a quantitative baseline for a continuous global synthesis, refining predictions of anthropogenic stressor impacts, identifying key research gaps and informing conservation strategies for freshwater ecosystems.

## Abstract (web summary)

This global meta-analysis of freshwater stressor–response relationships reveals that the biodiversity loss of five riverine organism groups reflects elevated salinity, oxygen depletion and fine sediment accumulation, while the relationship with nutrient enrichment and warming varies among groups.

## Main

Freshwater ecosystems—particularly rivers—have undergone rapid biodiversity declines in recent decades, driven by several interacting stressors across local, catchment and global scales^1,2^. Agricultural intensification, urban wastewater and sewer overflows degrade water quality^3–5^, while water abstraction exacerbates droughts and impervious surfaces intensify flash floods^6^. Other catchment-scale pressures, such as land reclamation, hydropower generation and navigation, further degrade habitat structure^7,8^. Meanwhile, global change further intensifies these impacts by disrupting flow regimes and thermal dynamics, compounding local pressures^9,10^. All these stressors may interact in complex ways, shaping the composition and diversity of riverine communities and making it challenging to predict biodiversity responses. Understanding these relations and the underlying mechanisms is crucial for effective conservation and management.

Over the past decade, research has increasingly focused on the cumulative effects of several stressors on riverine biodiversity. For example, Lemm et al.^11^ found that the ecological status of European rivers declines with increasing intensity of several stressors, while Brauns et al.^12^ demonstrated how several stressors impair ecosystem functioning. Experimental studies have explored how stressors interact—whether their effects are additive, synergistic or antagonistic—and investigated the underlying mechanisms^13–15^. However, despite substantial progress, a generalizable framework for predicting how several stressors collectively shape biodiversity remains elusive. This would require information, ideally raw data, on the relation between various organism groups and stressors from a range of ecoregions and river types, and a model that accounts for these multivariable data and the associated bias.

Paradoxically, the focus on multistressor impacts has obscured a critical knowledge gap: the absolute effects of individual stressors and their interactions with specific organism groups remain poorly quantified. Although numerous studies have examined individual stressor–response relationships^15^ and one global synthesis compared terrestrial, marine and freshwater communities^16^, a quantitative assessment across aquatic organism groups and stressors is still lacking.

Stressors affecting aquatic organisms can be broadly categorized as physicochemical stressors, which alter water quality and hydromorphological stressors, which modify habitat structure. Each stressor operates through distinct modes of action that may be caused by specific cellular mechanisms or through the provision/removal of habitats and that exclude or favour certain species. For instance, oxygen depletion slows metabolism^17,18^, while elevated salinity disrupts osmoregulation^19^. Hydromorphological changes, such as fine sediment accumulation or channelization, alter habitat availability, favouring some species while excluding others^20^.

The sensitivity of riverine organisms to these stressors varies widely. Larger organisms, such as fish and macrophytes, are disproportionately affected by habitat modifications including associated dispersal constraints^21,22^, while physicochemical stressors such as oxygen depletion and warming can affect a broader range of taxa^23,24^. Yet, systematic comparisons of stressor associations across different taxonomic groups remain rare^20,22^, limiting our ability to extrapolate localized findings to broader ecological contexts.

To address this gap, we present a global synthesis of stressor–response relationships across five key riverine organism groups—bacteria/archaea, algae, macrophytes, invertebrates and fish—focusing on seven widespread stressors. Drawing from 22,120 observational studies, we compiled 276 datasets encompassing 1,332 distinct stressor–response relationships. Each study sampled a riverine community at least six times (median = 14, mean = 58, s.d. = 346) focusing on at least one stressor (median = 3, mean = 3.3, s.d. = 2.5). We did not include experimental studies, as our prime interest lies in the relationship between stressors and biota under real-world conditions. Using multivariable generalized linear mixed models (GLMMs) and Bayesian meta-analyses, we quantified the associations of individual stressors and identified overarching response patterns.

This analysis provides a quantitative baseline for a continuous, global assessment of freshwater stressor impacts, enhancing our ability to predict biodiversity responses under increasing anthropogenic pressures. The prime objective was to establish empirical relationships between stressor intensity and biodiversity metrics over a wide range of conditions, independently of possible causes. Our findings offer crucial insights for conservation planning, informing mitigation strategies tailored to specific stressors and organism groups. By systematically quantifying stressor–response relationships, we contribute to a more predictive and actionable understanding of riverine ecosystem resilience in the face of accelerating environmental change.

## Results and discussion

### A global perspective on stressor–response relationships

**Fig. 1:**
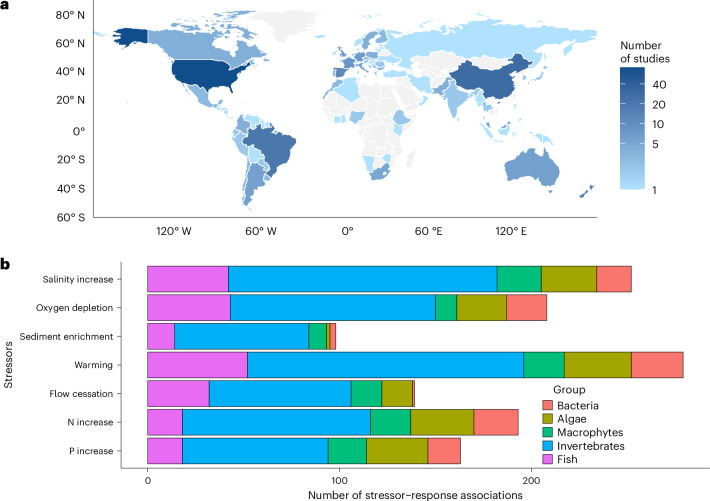
Data sources. **a**,**b**, Geographic distribution of data sources included in this synthesis (**a**) and breakdown of stressor categories by organism groups (**b**). Map data from Natural Earth (http://www.naturalearthdata.com).

Our meta-analysis revealed 1,332 stressor–response relationships from 276 studies across 87 countries (Fig. 1).

Nearly half the identified stressor–response relationships focused on invertebrates, with salinity and temperature being the most frequently studied stressors. In contrast, hydromorphological stressors—despite their recognized ecological importance^11^—were under-represented (Fig. 1), underscoring a critical research gap.

Taxonomic richness (for example, species or genus counts) that were analysed with log-linear models prevailed (92.9%), while for all logit-linear models evenness prevailed (71%) (Supplementary Table 4). In many individual studies, the relationships between stressors and biological responses varied greatly, often showing substantial heterogeneity. Although some stressor–response patterns are evident, the overall relations were relatively modest and highly variable for different stressors and organism groups. This highlights the inherent complexity of interpreting stressor–response dynamics in ecological systems (compare Supplementary Data 2).

### Relations of biodiversity patterns to single and combined stressor gradients

**Fig. 2:**
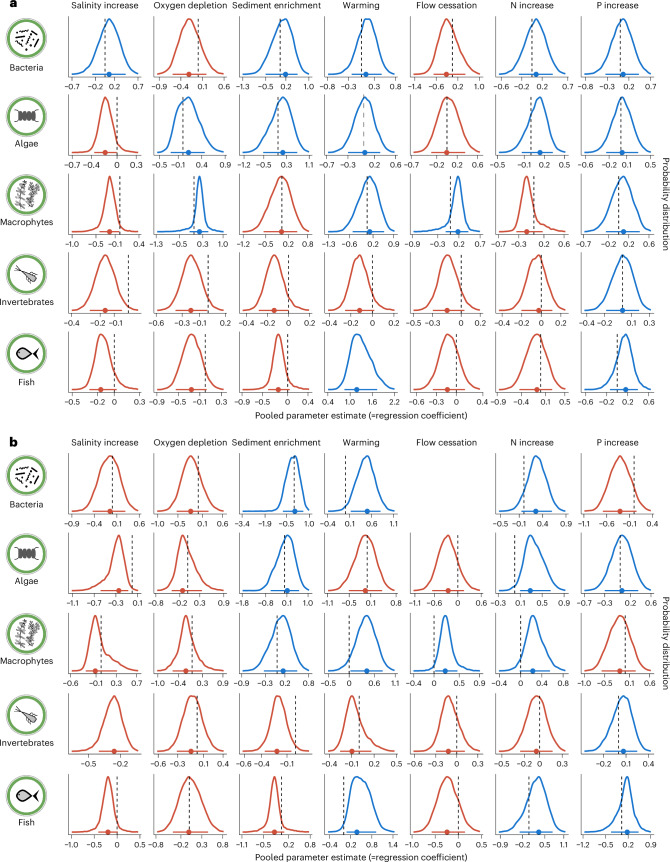
Stressor–response relationships. Posterior probability distributions (approximately the same as frequency of simulations) of regression coefficients for stressor–response relationships across five organism groups and seven stressors. **a**,**b**, Regression coefficients from log-linear models for taxonomic richness (**a**) and logit-linear models for evenness/coverage (**b**). Dots represent the mode, known as the MAP estimate; horizontal bars indicate 90% high-density intervals. Blue curves indicate positive mode estimates (approximately the same as positive response of organism group to stressor), while red curves indicate negative estimates. Full posterior results are given in Supplementary Data 2.

Only invertebrates consistently showed strong and negative relations to all stressors, except phosphorus enrichment, reflecting their dependence on oxygen availability, habitat structure and stable flow conditions^25–27^. In contrast to invertebrates, microorganisms—particularly bacteria/archaea—showed more variable patterns, probably due to their dependence on microscale conditions and the limited availability of suitable datasets^28–30^. Fish exhibited a mix of positive and negative stressor–response relationships, while relations of macrophytes to oxygen depletion and flow is opposing those of other groups, emphasizing their unique adaptations as sessile autotrophs (Fig. 2). However, our meta-analysis necessarily obscures divergent patterns of macrophytic taxa, such as bryophytes and vascular plants, which vary substantially in evolutionary origins, traits and environmental requirements.

Microbial responses require a dedicated sampling strategy to reflect microscale patterns. The response of bacteria/archaea was often divergent compared with macroorganisms, probably due to their environmental specificity and the under-representation of relevant studies. This includes methodological challenges to designate species and to assign operational taxonomic units that respond to stressor intensities. Notably, bacterial diversity exhibited a positive relationship with temperature, consistent with findings in marine systems^31–33^. Nutrient enrichment showed contrasting relations: bacterial diversity decreased with phosphorus—aligning with global patterns observed in lakes and streams^34^—but increased with nitrogen, probably due to the direct dependency of many taxa on nitrogen resources. Negative associations were frequently observed at higher nutrient concentrations, in the order of magnitude of 10 mg l^−1^ total nitrogen^35^, while the mean of all bacteria studies analysed here was 2.17 mg l^−1^ (s.d. = 0.8). Salinity and oxygen depletion showed no clear trends.

Algae, covering both planktonic and benthic algae, showed strong curve–linear relations to salinity and nutrient levels. The negative relationship to salinity was particularly strong, probably driven by osmotic stress^19,36^, supporting the general accepted conjecture that freshwater species are not directly replaced by brackish water species when salinity increases. Algal richness and evenness were positively associated with nutrient enrichment, particularly nitrogen, reflecting enhanced productivity^37^. This result contrasts with the general assumption that eutrophication is a prime stressor to phytoplankton biodiversity^35^—obviously, moderate nutrient input can enable higher alpha-diversity of algae. However, the more frequent occurrence of generalists and competitive species under eutrophic conditions may decline beta- and gamma-diversity because of disappearance of specialists for oligotrophic conditions^38^. The observed positive relationship may simply reflect higher specimen numbers in eutrophic waters, which increase the likelihood of detecting more species. Other stressor–response relations were weaker, suggesting indirect relations (for example, through another environmental variables) and data insufficiency.

Macrophytes (aquatic bryophytes and vascular plants) showed unique stressor–response patterns. They were positively associated with oxygen and flow cessation, probably benefiting from the stabilization of sediments and reduced force of current^39,40^. However, they showed negative relationships with salinity and nitrogen enrichment, which might favour a limited number of tolerant and competitive species outcompeting others and thus reducing species number and evenness^41,42^. These distinct associations highlight the specialized ecological niches occupied by macrophytes in river systems.

Invertebrates exhibited predominantly negative associations with both habitat and water quality stressors, particularly salinity, oxygen depletion, fine sediment accumulation and warming. These patterns probably reflect their sensitivity to habitat degradation and declining water quality^18,25,43^. Nutrient enrichment was only weakly associated, suggesting that direct nutrient impacts may be less relevant than habitat alterations and acts in an indirect way, for example through temporary oxygen depletion^20^.

Fish exhibit a mix of positive and negative stressor–response relationships. Negative relations were observed for salinity, oxygen depletion, sediment accumulation and flow cessation, reflecting habitat degradation and metabolic constraints^44^. Conversely, positive relationships with rising temperatures may reflect the dominance of warm-water species in downstream fish regions, which naturally exhibit a higher species richness compared with the cooler upstream areas; however, other factors such as habitat characteristics shape these patterns^45,46^. Nutrient enrichment showed minimal patterns and probably influenced fish only indirectly via oxygen depletion, habitat structure or prey availability.

**Fig. 3:**
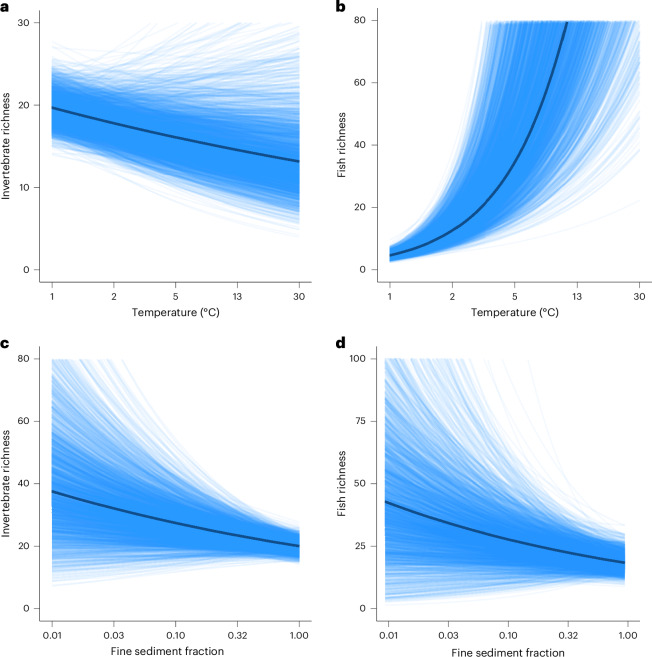
Hypothetical outcome plots. **a**–**d**, The HOPs predict taxonomic richness associations between invertebrates and temperature (**a**), fish and temperature (**b**), invertebrates and fine sediment (**c**) and fish and fine sediment (**d**).

Hypothetical outcome plots (HOPs) (Fig. 3) illustrate associations between taxonomic richness and selected key stressors, chosen on the basis of strength and uncertainty of observed relationships. Invertebrate and fish richness showed contrasting associations to increasing temperature (Fig. 3a,b), with a decline in invertebrate richness and a positive trend for fish richness. These patterns reflect differences in thermal sensitivity and physiological constraints. Invertebrate richness declined with warming, probably reflecting reduced oxygen availability. In contrast, fish richness tended to increase with moderate temperature rises, potentially coinciding with expanding river size naturally positively correlated with fish richness^47^ seasonal patterns^48^, as well as increase in exotic species^49^. Both, invertebrate and fish richness were negatively related to fine sediment accumulation and flow cessation, respectively (Fig. 3c,d), emphasizing the negative association between excessive sedimentation and reduced flow velocity on habitat complexity and potentially on oxygen availability. Fine sediment accumulation has been recognized as a key threat for riverine macroinvertebrates^50,51^ and fish^52^, but quantifications of stressor–responses relations remain scarce.

**Fig. 4:**
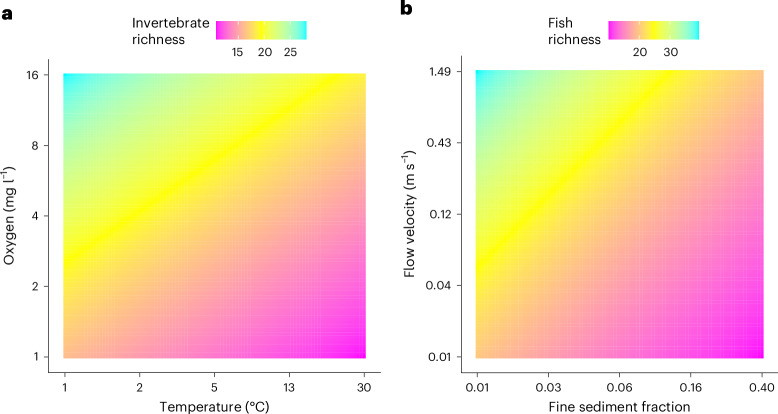
Partial dependence plots. **a**,**b**, The PDPs illustrate invertebrate richness (number of taxa) as a function of temperature and oxygen concentration (**a**) and fish richness (number of taxa) as a function of fine sediment enrichment and flow velocity (**b**), assuming additive stressor relationships.

Partial dependence plots (PDPs) (Fig. 4) illustrate the additive relation of two key stressors on taxonomic richness, highlighting how biodiversity related to several environmental stressors. Invertebrate richness as a function of temperature and oxygen depletion revealed that while high oxygen levels promote taxa richness, these benefits are reduced by elevated temperatures (Fig. 4a). Fish richness was consistently lower with higher fine sediment fraction and lower flow velocity (Fig. 4b), two hydromorphological stressors that are known to jointly reduce habitat quality and complexity. These findings reinforce the need for holistic management approaches, as the effectiveness of mitigating a single stressor may depend on the presence or severity of others that account for multistressor conditions rather than addressing stressors in isolation.

### Management and research implications

Our results emphasize the importance of management strategies tailored to regional conditions and stressor intensities^53^. Consistently, all taxa were strongly and negatively associated with salinity. As salinity is strongly correlated with other stressors, such as pesticides^54^, it may serve as a proxy for broader ecological degradation. Other stressors showed more variable associations. For example, warming was positively associated with the diversity of most organism groups, in particular fish, but negatively with invertebrates. At least in parts, this observation might simply reflect higher species numbers of warmer, downstream river reaches, which is most obvious for fish, while species richness of invertebrates typically declines downstream. These findings highlight the limitations of simplistic biodiversity metrics, such as species richness, which may obscure declines in specialist or functionally important taxa due to the proliferation of generalists. Despite these complexities, several overarching patterns emerged: salinization, oxygen depletion, fine sediment accumulation and flow cessation consistently related negatively with biodiversity, particularly for fish and invertebrates. In contrast, nutrient enrichment had minimal relations to most taxa but was strongly positively associated to algal diversity, although this observation might have been caused by the higher specimen numbers in samples from eutrophic waters potentially leading to reduced beta-diversity^38^.

Future research should aim to disentangle these relationships, identifying primary drivers to improve mitigation strategies. Addressing the observed variability in stressor–response patterns will require integrating local environmental conditions, species-specific tolerances and multivariable stressor interactions into ecological assessments.

Effective management of rivers must prioritize pollution reduction, sediment budget restoration and improved flow regimes to safeguard biodiversity. Additionally, fostering transparent data sharing and integrative modelling approaches will enhance our ability to refine stressor–response relationships and advance ecological understanding. By building on the findings of this study, future research can drive information-based conservation actions and support the sustainable management of aquatic ecosystems.

Beyond estimating the stressor–response relationships, this synthesis highlights the need for improved ecological data reporting. The available data reflect the research priorities of recent decades rather than the actual relevance of stressors, organism groups, or river types. The dataset is heavily dominated by studies from a few countries—particularly the USA, China and Germany—and macroinvertebrates are substantially better represented than other organism groups. In addition, several emergent stressors, such as contaminants and invasive species, were not considered because of difficulties in parameterization. In addition, small sample sizes and incomplete reporting constrained our ability to account for spatial and temporal autocorrelation, probably contributing to the heterogeneity among stressor–response relationships. Finally, the reliance on summary statistics (for example, means, medians and standard deviations) and the lack of accessible raw datasets constrain the precision of meta-analyses, a cumulative scientific approach, and hinder the detection of ecological patterns relevant to conservation and management. Greater availability of multivariate datasets, coupled with data-sharing practices, would enhance the transferability of findings. The dataset and analytical framework presented here provide a foundation for future assessments, offering an adaptable structure that can be updated using the accompanying code or by incorporating posterior estimates as priors in subsequent analyses. This flexibility supports ongoing monitoring efforts and ensures that findings remain relevant for guiding ecological research and management.

Bayesian approaches offer a promising avenue for synthesizing and integrating diverse data sources while accounting for parameter uncertainties^55^. Incorporating methods such as Bayesian network meta-analyses^56^ enables estimation of missing components by leveraging existing models, facilitating more robust predictions of stressor impacts. The compiled data source will act as a continuously improving baseline for the quantification of stressor–response relationships. Expanding predictive modelling capabilities through Bayesian methods can further refine stressor–response assessments, improving management strategies. These models can inform predictive-scenario testing, enabling managers to evaluate alternative stressor reduction strategies and their expected outcomes on biodiversity. Integrating such approaches into conservation planning will enhance our ability to mitigate anthropogenic stressors and strengthen freshwater ecosystem resilience.

## Methods

### Overview

We systematically identified studies that examined stressor–response relationships under field conditions. For each study, we extracted the data and fitted separate GLMMs to each dataset. The resulting parameter estimates (regression coefficients) from these individual models were then synthesized through a meta-analysis using Bayesian model averaging.

### Step 1: Data collection and literature search

**Fig. 5:**
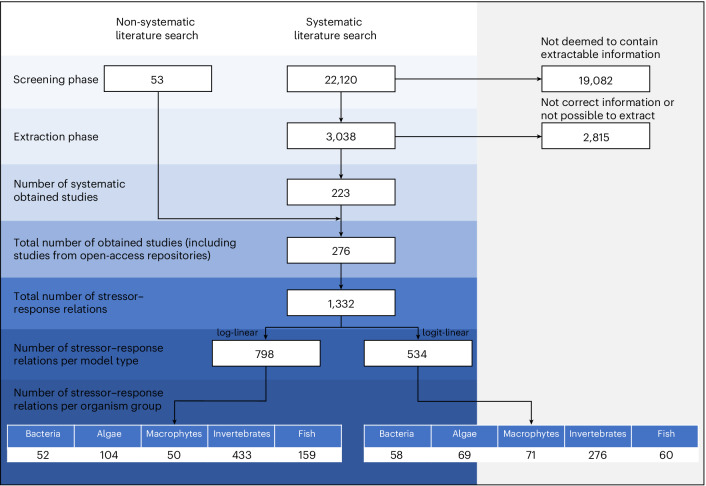
Article selection procedure. Flowchat depicting how the articles used in the meta-analysis were identified.

We compiled a comprehensive dataset on stressor–response relationships in riverine ecosystems under field conditions through a systematic Web of Science search. We encompassed five key riverine organism groups—bacteria/archaea, algae, macrophytes, invertebrates and fish—and seven major anthropogenic stressors—salinity increase, oxygen depletion, fine sediment enrichment, temperature increase, flow cessation and nitrogen and phosphorus enrichment. To ensure ecological realism, we excluded laboratory-based experiments, restricting our analysis to field studies. This search initially yielded 22,120 articles, which were systematically screened, resulting in 223 retained studies (Supplementary Information (step 1)). We supplemented this dataset with 55 additional studies from the open-access repositories Dryad, GitHub and Zotero, yielding a final dataset of 276 studies^22,25,57–330^ encompassing 1,332 quantified biota–stressor relationships (Fig. 5).

**Fig. 6:**
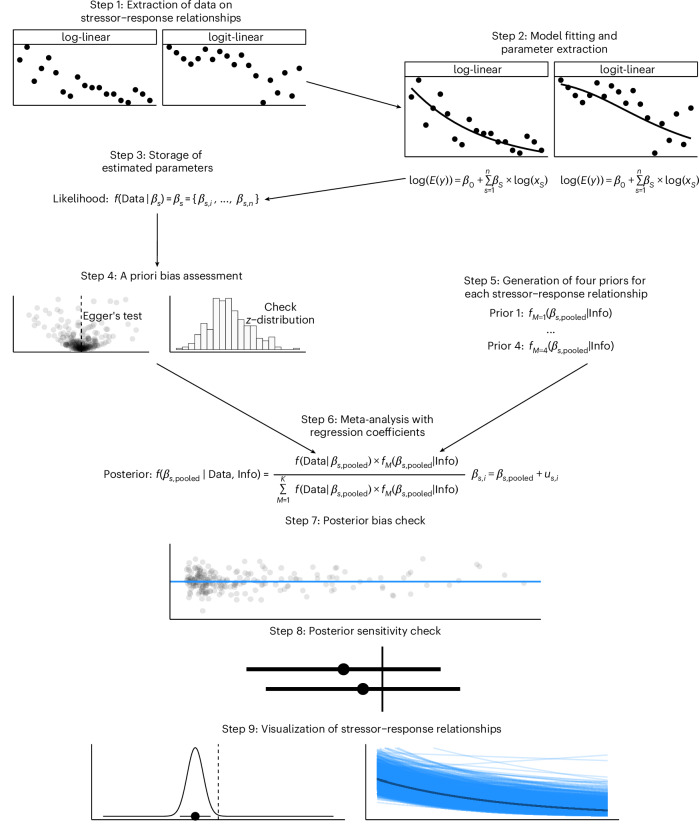
Analytical workflow. Steps for data extraction, model fitting, parameter storage, bias assessment, prior generation and meta-analysis.

We extracted data on key response variables, including taxonomic richness and evenness from figures, tables and supplementary datasets, prioritizing the most commonly reported metrics for each organism group. As independent variables, we extracted proxies of stressor intensities (for a full list of proxies compare Supplementary Information (step 1)). We also extracted if the study was based on a temporal or a spatial gradient (Fig. 6 (step 1) and Supplementary Information (step 1)). We refer to the data extracted from an individual study as ‘individual dataset’ in the following.

### Step 2: Model fitting and parameter extraction

The purpose of this step was to derive the model parameters and standard errors of the GLMM between each stressor and biotic response for each individual dataset, that is for each stressor–response relationship stemming from a single study. We applied GLMMs to model the response variables as follows. The linear component of the GLMMs was modelled with a log-link (taxonomic richness; count data) or with the logit-link (evenness and coverage; proportional data) (Fig. 6 (step 2)).

To facilitate parameter estimation, all independent variables were log-transformed (natural log), ensuring that model parameters corresponded to elasticity and semi-elasticity coefficients^331^. Elasticity coefficients, estimated via log-linear models, quantify the percentage change in a response variable per 1% increase in stressor intensity (for example, an elasticity of 0.2 indicates a 0.2% response change). Semi-elasticity coefficients, estimated via logit-linear models, measure the change in the logged odds of the response variable per 1% change in stressor intensity. When approaching zero, they closely approximate elasticity coefficients, enhancing interpretability.

This approach enables three key advantages: (1) the use of general priors across models, (2) comparable interpretation of stressor impacts and (3) the avoidance of self-referential issues and of bias introduced by *z*-transformations or minimum-maximum transformations, facilitating comparisons across models (Supplementary Information (step 2)).

If studies provided information on sampling dates, seasons or years, or on individual rivers sampled, random effects were applied if they did not lead to convergence issues in the model.

### Step 3: Storage of estimated parameters

All the estimated elasticity and semi-elasticity coefficients (regression coefficients and intercepts) for each stressor–response relationship were stored in a database (Fig. 6 (step 3) and Supplementary Fig. 2b (step 3)).

### Step 4: A priori bias assessment and quality control

Data extracted from literature might favour certain response categories, for example stronger and ‘significant’ over weaker ‘non-significant’ responses. To assess this potential publication bias, we applied Egger’s test, examining the shift in intercept based on the relationship between the inverse standard error and the ratio of parameter estimates to standard error (Fig. 6 (step 4)). Additionally, we analysed the distribution of *z*-values to identify systematic patterns indicative of selection bias^332^. These analyses revealed no clear bias, reinforcing the robustness of our estimates and minimizing the risk of overestimating stressor impacts (Supplementary Information (step 4)).

### Step 5: Prior formulation

A key element of our approach was the application of Bayesian model averaging (BMA) that allows for guiding models towards plausible stressor–response relationships based on prior information^55^. BMA requires the generation of several priors. In the Bayesian framework, posterior probabilities reflect updated priors of stressor–response relations given the likelihood of the data. To implement BMA, we generated four priors for each stressor–response relationship, classifying them into three sets: negative, neutral (unclear) or positive. This allowed us to incorporate directional expectations, such as the anticipated negative impact of salinity on freshwater biota. Details on prior selection are provided in Supplementary Information (step 5).

### Step 6: Meta-analysis

Applying the priors generated in step 5, we conducted a meta-analysis with the model parameters for each stressor–response relationship. We conducted a random-effects meta-analysis using BMA (Fig. 6 (step 6)) via R2JAGS. The Markov-chain-Monte-Carlo iterations were set to 30 chains with 3,000 iterations, thinned by 30. Model convergence was ensured by requiring: Rhat <1.01 and effective sample size >3,000. Bias adjustments were deemed unnecessary based on steps 4 and 7 (Supplementary Information (step 6)).

### Step 7: Posterior bias check

In addition to the a priori bias assessment (step 4) we conducted posterior bias checks following the meta-analysis (Fig. 6 (step 7)). We used funnel plots, which assessed posterior mean residuals as a function of the inverse of the standard error (1/s.e.). No diagonal patterns were observed, indicating no clear bias in stressor–response estimates (Supplementary Information (step 7)).

### Step 8: Posterior sensitivity check

We conducted a sensitivity analysis to evaluate the extent to which the priors influence the posterior estimates. To do this, we compared the posterior results presented in the main text with those from an alternative model using diffuse priors (priors without specific prior information) (Fig. 6 (step 8)). The analysis revealed that our prior assumptions about the stressor–response relationship tend to be more negative than the estimates derived from the data. Although some deviations from zero were observed, the estimated mode (the central tendency) remained stable (Supplementary Information (step 8)).

### Step 9: Visualization of stressor–response relationships

We generated several visualizations to enhance interpretability (Fig. 6 (step 9)). Posterior density plots (Fig. 2) illustrate plausible values for log-linear and logit-linear models, highlighting maximum-a-posteriori (MAP) estimates and 90% high-density intervals. HOPs (Fig. 3) visualize selected stressor–response combinations, modelling expected associations across stressor gradients while keeping other variables constant. Each HOP shows regression lines derived from posterior distributions, emphasizing variability. PDPs (Fig. 4) illustrate the marginal effects of two key stressors, aiding management-focused predictions (Supplementary Information (step 9)).

### Software and statistical packages

All analyses were performed in R. GLMMs were fitted with the glmmTMB package^333^ (v.1.1.8) that handles random-effect structures in ecological data. The GAMLSS package^334^ (v.5.4-20) was used for distributional analyses, while Bayesian modelling was conducted using JAGS (v.4.3.1) via the R2Jags package^335^. Data visualization was completed with ggplot2^336^ (v.3.4.4), cowplot^337^ (v.1.1.3) and bezier^338^ (v.1.1.2).

### Reporting summary

Further information on research design is available in the Nature Portfolio Reporting Summary linked to this article.

## Supplementary information

Supplementary Information Detailed description of the methodology.

Reporting Summary

Peer Review File

Supplementary Data 1 Details on data and priors.

Supplementary Data 2 Posterior estimations.

## Data Availability

Supplementary Data 1 provides details on all the articles and other data sources used in the meta-analysis and on the derived priors. Supplementary Data 2 lists the posterior estimations of all combinations of stressors and organism groups. Both files are available via GitHub at https://github.com/snwikaij/Data and Zenodo at 10.5281/zenodo.16947786 (ref. ^339^).

## Code availability

To support reproducibility, we developed an open-access R package, EcoPostView, enabling users to replicate, extend or visualize our analyses. This package allows custom data integration, alternative priors and posterior density visualization. The code and scripts used in this meta-analysis are available via GitHub at https://github.com/snwikaij/Data and the functions will be provided under the EcoPostView package via GitHub at https://github.com/snwikaij/EcoPostView.
