# Supplementary material for: Meta-analysis-derived estimates of stressor–response associations for riverine organism groups
Source: Nat Ecol Evol. 2025 Nov 11;9(12):2304–21. doi: 10.1038/s41559-025-02884-4 (PMC12680544; doi:10.1038/s41559-025-02884-4)
Supplement: Supplementary file 1 — Detailed description of the methodology. [file 41559_2025_2884_MOESM1_ESM.pdf]

nature ecology & evolution

Supplementary information https://doi.org/10.1038/s41559-025-02884-4

Meta-analysis-derived estimates of stressor–response associations for riverine organism groups

In the format provided by the authors and unedited

Table of content

Step 1 to 9 in Figure 6 are further explained in the supplementary material, providing a detailed description of each step.

- Step 1: Data sources and systematic search (Supplementary Text 1)
    - Supplementary Figure 1
    - Supplementary Table 1
    - Supplementary Table 2
    - Supplementary Table 3
- Step 2: Model fitting (Supplementary Text 2)
- Step 3: Accumulating estimates (Supplementary Text 3)
    - Supplementary Table 4
    - Supplementary Figure 2
- Step 4: A priori bias assessment and quality control (Supplementary Text 4)
    - Supplementary Figure 3
    - Supplementary Figure 4
    - Supplementary Figure 5
- Step 5: Prior formulation (Supplementary Text 5)
    - Supplementary Table 5
    - Supplementary Figure 6
- Step 6: Meta-analysis (Supplementary Text 6)
- Step 7: Posterior residual bias check (Supplementary Text 7)
    - Supplementary Figure 7
    - Supplementary Figure 8
    - Supplementary Figure 9
- Step 8: Sensitivity analysis (Supplementary Text 8)
    - Supplementary Figure 10
    - Supplementary Figure 11
    - Supplementary Figure 12
- Step 9: Visualizing the results (Supplementary Text 9)
- References

Step 1: Data sources and systematic search

A systematic literature search was conducted on March 5, 2024, using Web of Science. The search terms targeted five key organism groups (bacteria/archaea, algae, macrophytes, invertebrates, and fish) and six major stressors (oxygen depletion, salinity, fine sediment, flow changes, nutrient enrichment, and temperature increase). The search string was as follows:

phytoplanct* OR macrophyt* OR fish* OR piscine* OR macroinvert* OR benthic Invert* OR invertebrate* OR diatom* OR algae* OR phytobenth* OR bacteria* OR aquatic vegetation* OR aquatic plant* OR submerged vegetation* (Topic) and salini* OR conductiv* OR salt* OR oxygen* OR fine sediment* OR flow* OR velocit* OR discharge* OR temperature* OR thermal* OR nutrient* OR nitrat* OR nitrogen* OR phosph* (Topic) and freshwater OR stream OR river (Topic) not marine* OR lake* OR pond* OR ocean* OR sea* OR terrestrial* OR mammal* OR fungi* OR labor*

This search retrieved 22,120 articles (Fig. S2). It was designed to exclude studies on laboratory experiments. Each researcher screened approximately 1,000 articles, selecting those that reported on taxon richness, community composition, evenness, Shannon Index, or proportion of sensitive species (e.g., % Ephemeroptera, Plecoptera, and Trichoptera - EPT) within the focal organism groups. To enhance inclusivity, coverage (%) was also considered for macrophytes. Only field-based studies were included, ensuring ecological relevance.

The six selected stressors were quantified using the following proxies:

- Oxygen: Oxygen concentration (mg L^{-1}),
- Salinity: Conductivity (µS cm^{-1}) or chloride concentration (mg L^{-1}) / 0.6
- Sediment: Fraction of fine sediment (%), fine sediment % = 1-coarse sediments (%), silt + sand (%), silt (%), sand (%), or mesolithal (%) as a proxy for substrate fines.
- Warming: Temperature (°C)
- Flow cessation: Flow velocity (m s^{-1}) or discharge (m^3 s^{-1})
- Nutrient-N: Total nitrogen (mg L^{-1}), nitrate (mg L^{-1}), dissolved inorganic nitrogen (mg L^{-1}), or nitrate+nitrite (mg L^{-1}).
- Nutrient-P: Total phosphorus (mg L^{-1}), phosphate (mg L^{-1}), soluble reactive phosphorus (mg L^{-1}), or dissolved inorganic phosphorus (mg L^{-1}).

Before screening, false-negative rates were assessed using a pre-screened set of 33 articles (14 containing relevant data), which were given to each analyst to evaluate their ability to detect extractable data from figures, tables and supplements. The false-negative proportion, i.e. the rate of relevant articles the analysts overlooked, was 0.23 (SD = 0.15, n = 23). To mitigate bias, analysts received feedback on errors from the first author before proceeding with their assigned articles. A skew-normal prior was applied to reflect a conservative bias toward lower false-negative rates [SkewNormal(0.05, 0.5, 12); T(0,1)], yielding a posterior mean of 0.23 with reduced variance (SD = 0.13). The estimated false-negative range within 90% high-density intervals (HDIs) was 4-43% (Fig. S1).

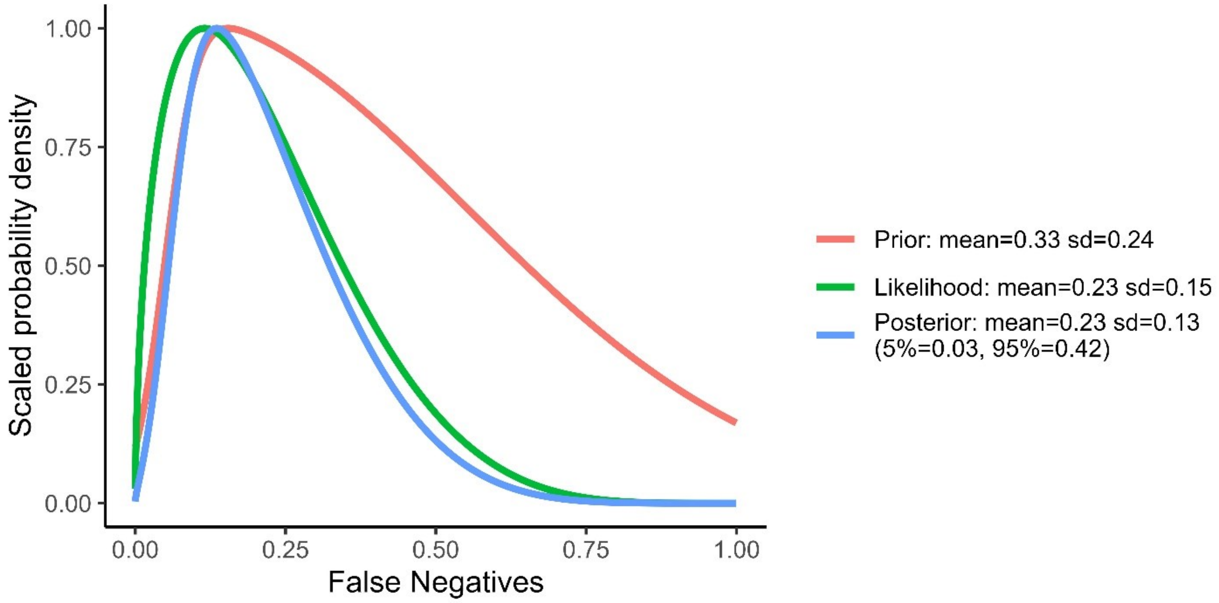

Supplementary Figure 1: Prior distribution for false-negative estimation using the sn package (v2.1.1) in R. Likelihood estimation used glmmTMB (v1.1.8) with an intercept model and zero-inflation.

The following inclusion criteria were applied:

- Only datasets with n > 5 observations were considered.
- Supplementary data not embedded in the article were excluded due to infeasibility of manual retrieval.
- Studies were only considered if biodiversity metrics were reported (evenness, Shannon diversity, number of species, genus, family or order, abundance).
- Mean or median values were only extracted when explicitly provided.
- Aggregated community matrices were considered unless data extraction was unreliable (e.g., [1])

The data extraction was conducted by seven researchers. When samples exhibited temporal variation - such as seasonal, monthly, or yearly differences - reflected in figures, tables, or datasets, these temporal factors were included as random effects, provided model convergence permitted. The majority of studies reporting a temporal gradient based on samples collected between March and November, with June having the highest sampling density (Tab S1).

Supplementary Table 1: Distribution of samples taken per month.

	Jan	Feb	Mar	Apr	May	Jun	Jul	Aug	Sep	Oct	Nov	Dec
Observations	9	11	26	23	29	42	39	36	31	33	16	13

Most sampling periods occurred within a single year. Sample years per study were 2 (mean) and 1 (median), respectively (Standard deviation = 3).

The number of samples per group and stressor varied considerably (Tab S2). For example, bacteria and N-increase had 22 samples at 95% quantile and macrophytes and salinity-increase samples 989 at 95% quantile

Supplementary Table 2: Number of observations per combination of organism group and stressor.

Organism group	Stressor	Mean	Median	Standard Deviation	5%	95%
Bacteria	Salinity-increase	17	11	16	8	33
Bacteria	Oxygen-depletion	14	8	15	8	33
Bacteria	Sediment-enrichment	33	12	37	12	70
Bacteria	Warming	16	9	14	7	30
Bacteria	Flow-cessation	76	76	NA	76	76
Bacteria	N-increase	14	8	14	8	22
Bacteria	P-increase	15	8	17	8	33
Algae	Salinity-increase	74	16	201	6	291

Organism group	Stressor	Mean	Median	Standard Deviation	5%	95%
Algae	Oxygen-depletion	28	16	37	6	88
Algae	Sediment-enrichment	98	98	2	96	99
Algae	Warming	70	18	183	6	236
Algae	Flow-cessation	37	11	48	6	117
Algae	N-increase	58	16	182	6	132
Algae	P-increase	68	16	185	7	158
Macrophytes	Salinity-increase	135	15	378	10	989
Macrophytes	Oxygen-depletion	24	14	32	12	71
Macrophytes	Sediment-enrichment	29	15	35	13	82
Macrophytes	Warming	23	15	24	8	32
Macrophytes	Flow-cessation	52	14	126	6	219
Macrophytes	N-increase	96	15	252	8	515
Macrophytes	P-increase	103	18	257	8	544
Invertebrates	Salinity-increase	82	15	582	6	109
Invertebrates	Oxygen-depletion	31	15	42	6	116

Organism group	Stressor	Mean	Median	Standard Deviation	5%	95%
Invertebrates	Sediment-enrichment	43	17	80	6	215
Invertebrates	Warming	33	14	72	6	96
Invertebrates	Flow-cessation	33	12	62	7	137
Invertebrates	N-increase	33	14	73	6	110
Invertebrates	P-increase	27	12	37	6	111
Fish	Salinity-increase	18	12	17	6	63
Fish	Oxygen-depletion	18	12	21	6	68
Fish	Sediment-enrichment	23	13	23	6	71
Fish	Warming	44	12	153	6	118
Fish	Flow-cessation	43	16	47	8	112
Fish	N-increase	31	12	56	8	97
Fish	P-increase	79	12	254	8	225

Supplementary Table 3: Number of datasets for combinations of organism groups and stressors. Only studies with six or more observations (n > 5) were included.

Group	Salinity increase	Oxygen depletion	Fine sediment increase	Temperature increase	Flow cessation	N increase	P increase	Sum
Bacteria	18	21	3	27	1	23	17	110 (8.3%)
Algae	29	26	2	35	16	33	32	173 (13%)
Macro-phytes	23	11	9	21	16	21	20	121 (9.1%)
Inverte-brates	140	107	70	144	74	98	76	709 (53.2%)
Fish	42	43	14	52	32	18	18	219 (16.4%)
	252 (18.9%)	208 (15.6%)	98 (7.4%)	279 (20.9%)	139 (10.4%)	193 (14.5 %)	163 (12.2%)	1332 (100%)

Response variables

We extracted count data or proportional metrics. Count data included the number of orders, families, genera or species (one discrete response per study).

Proportional metrics included evenness that was either extracted directly or calculated as Evenness = Shannon / log(taxa number). If evenness exceeded 1 (due to summary statistics), a pseudo-evenness metric was applied: Pseudo-evenness = Shannon / max(Shannon). Other proportional metrics that were used if data on evenness were unavailable included % EPT-taxa (invertebrates) and vegetation coverage (%) (macrophytes), the latter was calculated as coverage % = bryophytes % + vascular plants %.

The full list of all extracted data is provided in Supplementary Data 1. The function provided within the R-package EcoPostView can explore any desired specific model results.

Data sources were categorized as “figure,” “table,” or “dataset” to assess bias, i.e. if stronger stressor-response relationships were observed for data presented in figures and tables compared to data given in the annexes (Step 5). If data originated from multiple sources, the dominant category was assigned.

Step 2: Model fitting and parameter extraction

We fitted a Generalized Linear (Mixed) Model (GLM(M)) to each individual dataset, with log- or logit-links using Negative Binomial or Beta distributions to estimate stressor-response relationships. If response variables contained excessive zero or one values, zero-inflated or zero-one inflated components were included.

Model structure

- Random effects: Variance across groups due to different clusters (e.g., rivers, months, years, seasons) was modeled as a random effect, unless convergence issues arose.
- Optimization: If convergence failed, we switched from nlminb (default) to BFGS (Broyden-Fletcher-Goldfarb-Shanno algorithm). If issues persisted, first random effects were dropped, then if needed the stressors were removed in the following order: salinity → oxygen → nutrient-N → nutrient-P → fine sediment → temperature → flow (based on a priori heuristic-informed interpretation of stressor prevalence).
- Residual checks: The DHARMa package[2] was used to generate residual plots, considering only extreme deviations as suspicious.

To ensure comparability, all independent variables were log-transformed (Ln). Using log- and logit-link functions, estimated parameters represent elasticity or semi-elasticity coefficients:

- Elasticity coefficient (log-link model): $\beta 1 = \frac{d\,Log(y)}{d\,Log(x)}$ A coefficient of 0.2 indicates a 0.2% change in the response variable per 1% change in stressor intensity.
- Semi-elasticity coefficient (logit-link model): $\beta 1 = \frac{d\,logit(y)}{d\,Log(x)}$ This approach enables comparison across taxonomic levels and stressors.

Example calculation

Consider a simple linear model:

- Family richness: y1={1, 7}, Species richness: y2={10, 70}
- Stressor gradient: x={25, 350}

$$coefficient = \beta_1 = \frac{7-1}{25-350} = -0.185 \text{ and } \beta_1 = \frac{70-10}{25-350} = -0.0185$$

$$elasticity\ coefficient = \beta_1 = \frac{Ln(7)-Ln(1)}{Ln(25)-Ln(350)} = -0.737 \text{ and } \beta_1 = \frac{Ln(70)-Ln(10)}{Ln(25)-Ln(350)} = -0.737$$

The elasticity coefficient allows us to express the percent (%) change in the response variable per percent (%) change in the stressor [3]. For example, an elasticity coefficient of 0.2 indicates a 0.2% increase in the response per 1% increase of the stressor. This approach eased the interpretation between and within groups. Since families and species exhibit similar responses,

taxonomic level selection does not affect β_1, facilitating stressor-response comparisons. Furthermore, priors can be easily formulated based on comparison to extremely strong relations. Finally, the ratio log(y unit)/log(x unit) maintains unit interpretability on the log scale, allowing for consistent comparisons across studies.

In contrast, we avoided z-standardization and min-max transformations because they eliminate unit interpretability and introduce scaling-dependent distortions, which can increase model error [4]. This ensures the estimated parameters remain comparable beyond the study, which is not possible after scaling and transformation.

In fact, such transformations hinder comparability across studies. For example, with z-standardization, "*coefficient estimates may not necessarily be comparable across studies that use different samples.*" [5], since standardization depends on sample-specific means and standard deviations.

To illustrate, suppose Study A has a mean of 500 and standard deviation (SD) of 300, and Study B has a mean of 150 and SD of 50. If a new observation is 300, then under z-standardization:

$$Study\ A: = \frac{(300-500)}{300} = -0.67$$

$$Study\ B: = \frac{(300-150)}{50} = 3$$

Despite the raw value being identical, the standardized values differ drastically, introducing variability and reducing precision in parameter estimation.

Min-max transformation similarly assumes a consistent data range across studies. For example, for oxygen measured in mg/L:

$$Study\ A: 5 - 12\ mg \cdot L^{-1} = \frac{(9-5)}{12-5} = 0.57$$

$$Study\ A: 8 - 12\ mg \cdot L^{-1} = \frac{(9-8)}{12-8} = 0.25$$

Again, the same raw value (9 mg/L) is mapped to different scaled values, resulting in non-comparable model parameters.

In contrast, log-log $\beta_1 = \frac{log(y)}{log(x)}$ or logit-log $\beta_1 = \frac{logit(y)}{log(x)}$ transformations are scale-invariant and preserve the structure and units of the original data, making model estimates more interpretable and comparable across different datasets.

Step 3: Storage of estimated parameters

Point estimates for the intercepts (β_0) and regression coefficients (β_i) of log- and logit-linear models (*g*) were extracted from each fitted model, along with standard errors (SE):

$$g(E(y_i|x_{ij})) = \beta_0 + \sum_{j=1}^{j}(\beta_j \cdot Log(x_{ij})).$$

We recorded the organism group, stressor type, and model type (link function and error distribution; see Supplementary Data 1). The response variables used are broadly categorized in Tab. S4. Fig. S2 displays the elasticity (a) and semi-elasticity coefficients (b) extracted from all fitted models.

Supplementary Table 4: Categories of response variables used in this study.

	**Discrete response (Log-linear)**				**Proportional response (Logit-linear)**				
**Organism group**	Taxon	Sensitive taxon	Sensitive abundance		Even-ness	pseudo-evenness=shannon/max(shannon)	Fraction sensitive	Coverage	
**Bacteria/ Archaea**	52	0	0	52 (6.5%)	42	16	0	0	58 (10.9%)
**Algae**	104	0	0	104 (13%)	44	20	5	0	69 (12.9%)
**Macrophytes**	50	0	0	50 (6.3%)	11	4	0	56	71 (13.3%)
**Invertebrates**	378	39	16	433 (54.3%)	151	32	93	0	276 (51.7%)
**Fish**	159	0	0	159 (19.9%)	44	15	1	0	60 (11.2%)
	743 (92.9%)	41 (5.1%)	16 (2%)	800 (100%)	292 (54.7%)	87 (16.3%)	99 (18.5%)	56 (10.5%)	534 (100%)

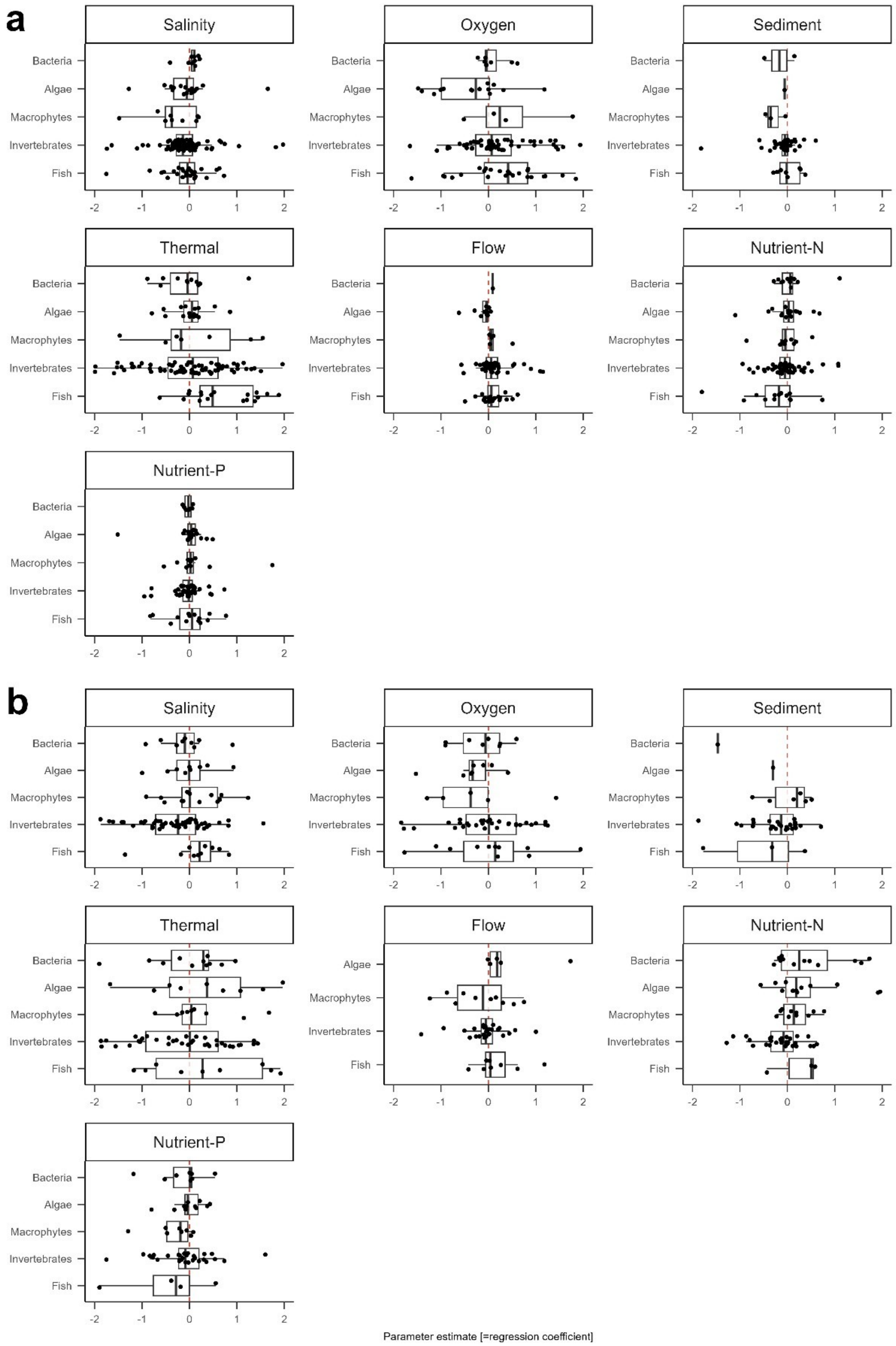

Supplementary Figure 2: Point estimates for the (a) log-linear and (b) logit-linear models. X-axis limits are set to -2 and 2 for visibility.

Step 4: A priori bias assessment and quality control

Prior to the meta-analysis, we assessed potential publication bias using two methods. This step was performed to test if data extracted from literature favour certain response categories, e.g. stronger over weaker responses.

Regression-based bias detection (Egger's Test)

We examined the relationship between precision (1/SE) and standardized parameter estimates (parameter estimate/SE). A significant shift in the intercept suggests publication bias, where higher standardized estimates are associated with larger SEs. This relationship was tested using a linear mixed model (LMM), commonly known as Egger's test.

The source (figure, table, or dataset) was modeled as a random intercept, nested within study ID. We examined whether a particular source biased stressor-response relationships by modeling the regression coefficient for each source as a random effect. The source is potentially relevant as we aimed to test if stronger stressor-response relationships were observed for data presented in figures and tables compared to data given in the annexes.

Priors for Bias Assessment:

- The alternative model (*M1*) assumes potential bias, modeled as a mixture of two normal distributions *N*(-0.2, 0.1) and *N*(0.2, 0.1) (red curves in Fig. S3).
- The null model (*M0*) assumes no bias, modeled as *N*(0, 0.025) (blue curve in Fig. S3).

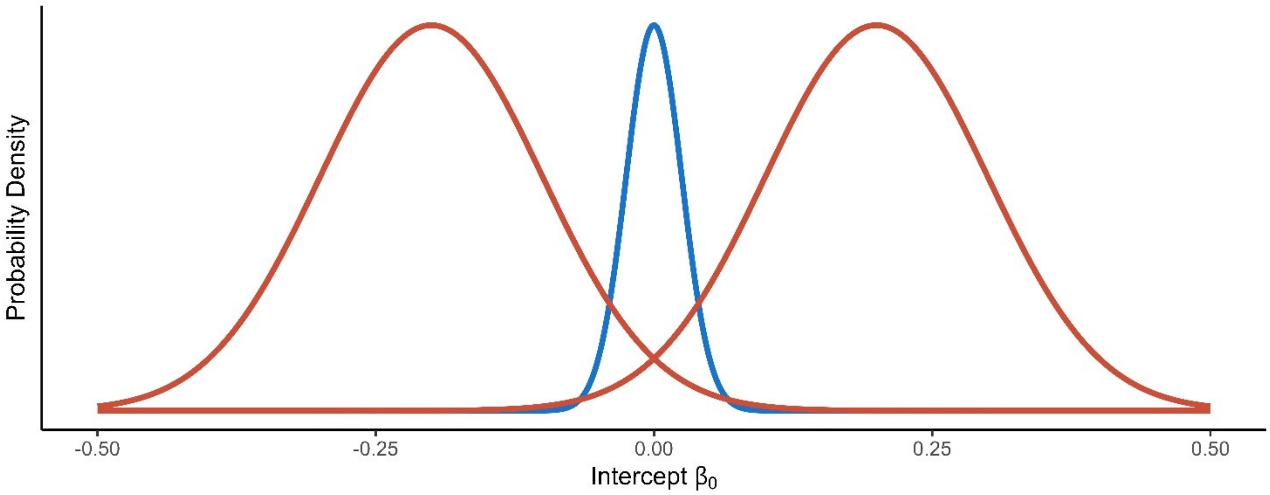

Supplementary Figure 3: Visual example for M1 assuming potential bias (red curves) and M0 assuming no bias (blue curve).

The slope (β_1) was assigned a vague prior *N*(0, 1), and the model's standard deviation was set as *U*(0, 10). The Bayes Factor (BF_{10}) represents how much the data favors *M1* over *M0* (Fig. S4).

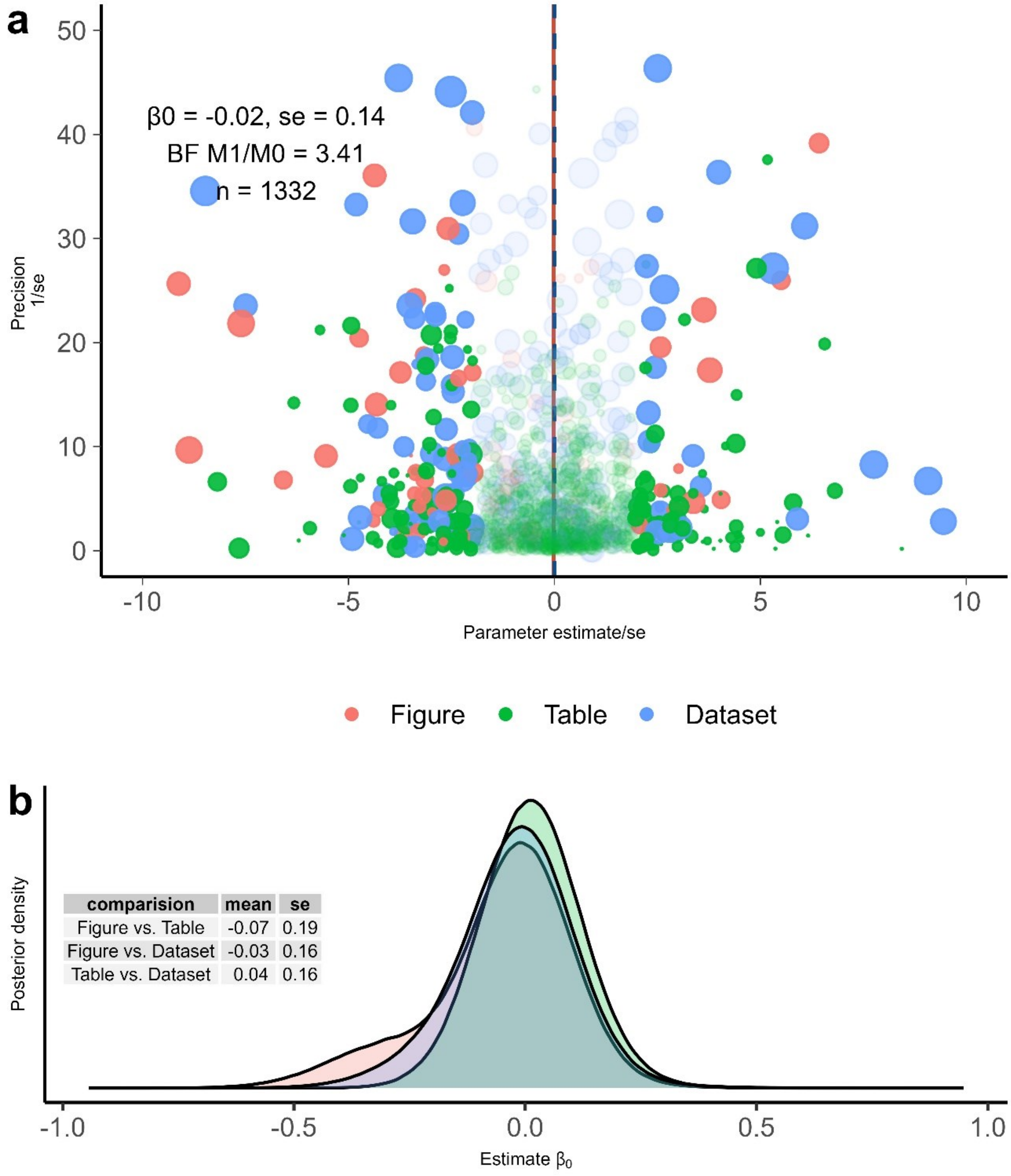

Supplementary Figure 4: (a) Funnel plot of 1/SE (y-axis) vs. parameter estimate/SE (x-axis). The solid red line represents the expected value regression line; the dashed blue line represents the intercept-only model. Ideal conditions would result in overlap. Point size reflects sample size, point color indicates data source type (figure, table, or dataset), and transparency corresponds to the z-score (transparent for |Z| < 1.96 or p > .05). Symbols: β_0 = intercept, BF = Bayes Factor, n = sample size, and se = standard error of β_1. (b) Estimates of the mean intercept difference for each data source in a linear mixed model.

Z-Value distribution analysis

We evaluated the distribution of z-values (z = estimate / SE) across stressors. Ideally, this distribution should approximate a normal distribution with no noticeable gaps around -1.96 and 1.96, as reported by [6]. In short, since the estimate could, in theory, take any value from $-\infty$ to ∞ and the standard error (SE) must be greater than 0, the possible values for z=estimate/SE also range from $-\infty$ to ∞. According to the central limit theorem, as the sample size within each study increases, the sampling distribution of the individual study estimates tends to approximate a normal distribution. Thus, we can model the estimate as approximately following a normal distribution. $N(\mu,SE)$, where μ represents the (unknown, fixed or random) underlying population mean and SE is the standard error of the estimate. When we standardize the estimate by subtracting μ and dividing by its standard error, we obtain the standardized statistic: $Z=(\text{estimate}-\mu)/SE$. If we assume $\mu=0$ then it simplifies to $Z=\text{estimate}/SE$. Under these assumptions, the Z-statistic approximately follows a standard normal distribution.

Fig. S5 shows z-value distributions across stressors, with vertical red dashed lines indicating -1.96 and 1.96.

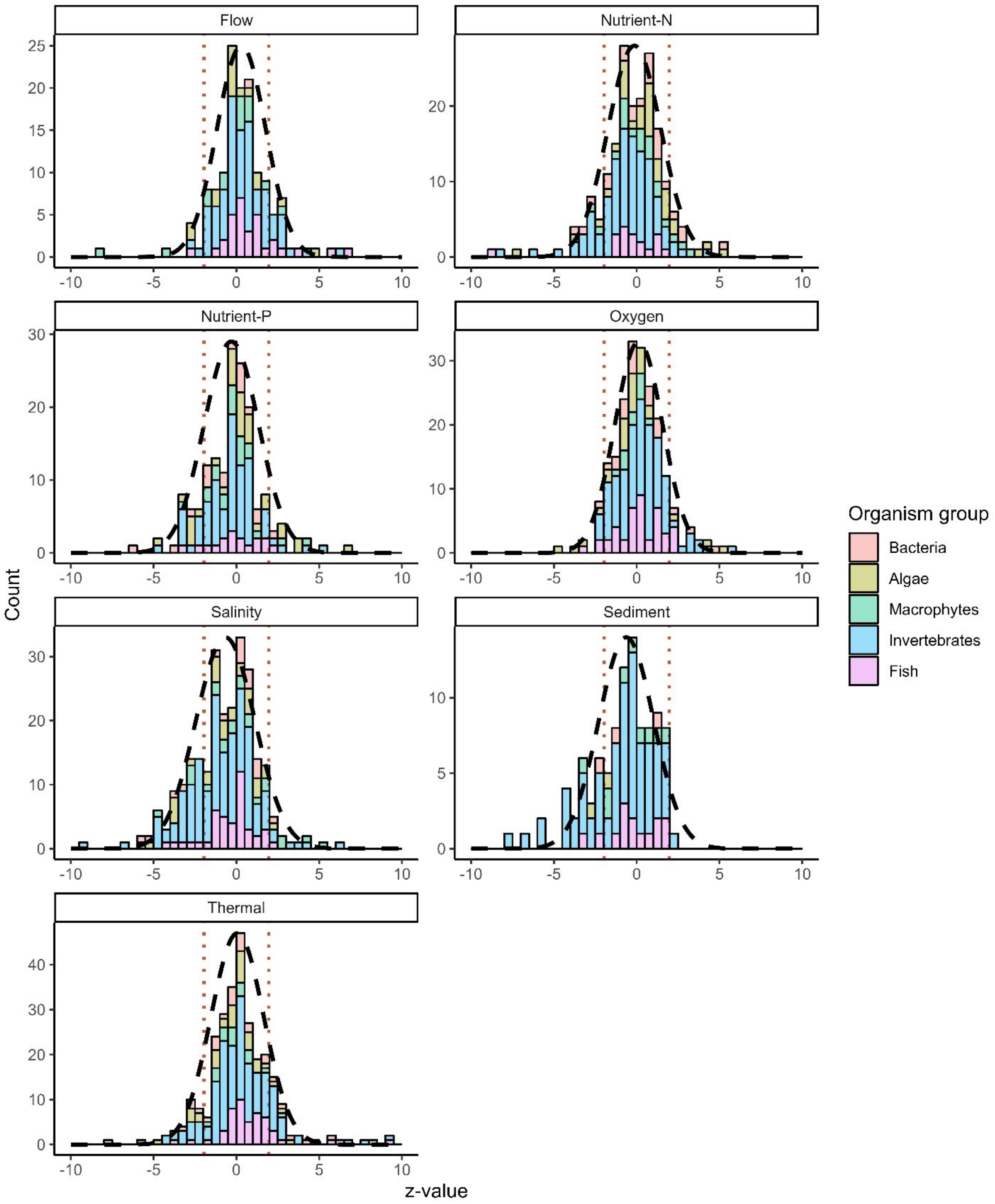

Supplementary Figure 5: Distribution of z-values derived from the observed parameter and standard error for each stressor. The biotic groups are given as colors.

Step 5: Prior formulation

A key element of our approach was the application of Bayesian Model Averaging (BMA) that allows for guiding models toward plausible stressor-response relationships based on prior beliefs. BMA requires the generation of multiple priors.

The posterior distribution β_{pooled} of the fitted models from Step 3 was estimated using Markov Chain Monte Carlo (MCMC) via the R2JAGS package in R [7]. The MCMC is a simulation method to update our prior information after observing the likelihood of the posterior probability $\beta_{[stressor-response,link]}$, given the estimations (β). Posterior ∝ Likelihood * Prior:

$$\beta_{[stressor-response,link]} = \{\beta_{[stressor-response,link,study]}, \cdots, \beta_{[stressor-response,link,study]}\}$$
$$P(\beta_{[stressor-response,link]}|Data, Info) = P(Data|\beta_{[stressor-response,link]}) \propto P(\beta_{[stressor-response,link]}|Info)$$

This can be simplified to

$$P(\beta|Data, Info) = P(Data|\beta) \propto P(\beta|Info)$$

where the prior *P(β|Info)* encodes our current understanding of the stressor-response relationship, reducing weight for implausible values and increasing weight for reasonable ones. Consequently, this Bayesian approach does not focus on detecting 'effects' in the frequentist sense, but rather refines prior knowledge about the parameter (β).

More formally, we seek to determine the most probable values for β given the available data and prior information $P(\beta|Data, Info)$. This approach solely evaluates the relationship between data and prior information through the posterior distribution [8,9].

Unlike frequentist methods, it does not assess statistical significance in terms of p-values. Specifically, frequentist inference tests whether the probability of observing the data is greater than or equal to zero, given the null hypothesis is true:

$P(Data \geq 0|\beta = 0)$

In contrast, this Bayesian inference provides a probabilistic estimate of β while incorporating prior knowledge, leading to a more nuanced understanding of stressor-response relationships.

Prior specifications

Our use of priors serves two main purposes:

1. To incorporate a range of plausible ecological expectations, based on existing notions of "diffuse", "ignorant", "directional", and "strong" informative priors.

2. To adhere to the principles of Bayesian inference, where not specifying priors is essentially neglecting the core tenet of Bayes' theorem - that prior information should

inform the analysis.

We did not rely on a single prior. Instead, we explored four different priors, ranging from weakly to moderately informative:

- A Diffuse prior (uninformative prior) Normal(0, 10).

- An Ignorant prior, Normal(0, 0.5), was used to reflect minimal prior knowledge regarding its range.

- An informative prior Normal(-0.3, 0.3) or N(0.3, 0.3) that reflects widespread ecological expectations based on information from literature - for example, the notion that increasing salinity tends to reduce freshwater invertebrate diversity at broad scales.

- A Strong prior Normal(-0.2, 0.1) and N(0.2, 0.1) that reflects widespread ecological beliefs and salinity tends to reduce freshwater invertebrate diversity at broad scales.

Our approach does not constrain the analysis to only positive or negative stressor-response relationships. Rather, it embraces a range of ecologically plausible scenarios and allows the data to update and refine this prior information.

Using Bayesian model averaging, we allow each prior to contribute to the posterior distribution. This is done under the assumption of equal but stochastic prior weights, modeled via a Dirichlet distribution.

Diffuse prior

A diffuse prior allows for a broad range of values and was set as a normally distributed with a mean of 0 and a standard deviation of 10: $N(0,10)$. This was the only prior applied to the intercepts.

Ignorant prior

Based on well-established relationships, and strong relation such as chlorophyll-a versus total phosphorus [10–12], values typically range from 1 to 1.5 in log-log or log10-log10 models [13–21]. Similar to the relationship described by [22], these values remain within the boundaries of ~-1.5 and ~1.5 when stressor gradients are square root transformed. Therefore, an upper and lower bound of -1.5 and 1.5 was selected as at 99% $N(0, 0.5)$.

Directional priors

These priors were informed by literature and calculated elasticity coefficients, following a structured three-step approach:

Step A: [23] estimated a decline of macrophyte richness and chlorophyll-a of -0.32 in a Log-Log model, a weaker but still clear relationship compared to the chlorophyll-a versus total phosphorus correlation. [24] reported an average Logit~Log regression coefficient for conductivity centered

around -0.3. Similarly, [25] found that, in a log-linear model, regression coefficients for conductivity and total nitrogen were -0.27 and -0.41, respectively, for EPT species count. Oxygen, which was not log-transformed, exhibited a positive coefficient of 0.23.

Step B: Literature estimates of species richness boundaries were used to define expected maximum and minimum declines. For instance, species richness of invertebrates in brackish water ranges from approximately 10 to 30 species [26,27], whereas freshwater rivers can support up to 60 to 80 species [28]. Using elasticity coefficient equations, prior expectations were formalized. The maximum expectation considered a decline from 80 to 10 species:

$$\beta_{strong} = \frac{Ln(80) - Ln(10)}{Ln(50) - Ln(5000)} = -0.45$$

And a minimum decrease in species richness from 60 to 30:

$$\beta_{strong} = \frac{Ln(60) - Ln(30)}{Ln(50) - Ln(5000)} = -0.15$$

This approach was applied to each organism group and stressor. The numbers in Tab. S5 for species richness were based on expert judgment and the following figures and tables from literature [28–40]. The average of all values in Tab. S5 is -0.37.

Step A results in a mean of 0.31 from the absolute values (0.32, 0.3, 0.27, 0.41, 0.23), which is in the same order of magnitude as Step B. Therefore, the mean of a directional prior is either -0.3 (negative) or 0.3 (positive).

Supplementary Table 5: Estimation of an average prior relation of multiple stressor-response relation.

	Lower		Upper								
	minimum	maximum	minimum	maximum	Salinity	Oxygen	Sediment	Thermal	Flow	Nutrient-N	Nutrient-P
					50	4	0.01	4	0.01	1	0.01
					5000	16	1	25	1.5	30	1.5
Bacteria	6000			9000	-0.088	-0.292	-0.088	-0.221	-0.081	-0.119	-0.081
Bacteria		7000	8000		-0.029	-0.096	-0.029	-0.073	-0.027	-0.039	-0.027
Algae	20			100	-0.349	-1.161	-0.349	-0.878	-0.321	-0.473	-0.321
Algae		30	60		-0.151	-0.500	-0.151	-0.378	-0.138	-0.204	-0.138
Macrophytes	3			15	-0.349	-1.161	-0.349	-0.878	-0.321	-0.473	-0.321
Macrophytes		5	10		-0.151	-0.500	-0.151	-0.378	-0.138	-0.204	-0.138
Invertebrates	10			80	-0.452	-1.500	-0.452	-1.135	-0.415	-0.611	-0.415
Invertebrates		30	60		-0.151	-0.500	-0.151	-0.378	-0.138	-0.204	-0.138
Fish	5			20	-0.301	-1.000	-0.301	-0.756	-0.277	-0.408	-0.277
Fish		3	15		-0.349	-1.161	-0.349	-0.878	-0.321	-0.473	-0.321

Step C: The standard deviation was derived from variation from the effect-sizes across three meta-analyses[41–43], two independent studies [44,45], Tab S5 above and a personal dataset and evaluating multiple models relating planktonic and benthic chlorophyll-a to total phosphorus. These sources provide a robust summary of known variability in stressor-response relationships.

To ensure consistency, all effect sizes were converted to Fisher's z, placing them on the same scale. The standard deviation was then individually calculated from the five published studies and additional datasets ([41] = 0.52, [42] = 0.34, [43] = 0.07, [44] = 0.31, [45] = 0.23, Supplementary Table 5 = 0.32, personal dataset = 0.38). The average standard deviation from these seven values was 0.31, rounded to 0.3.

This resulted in two directional priors with means of -0.3 or 0.3 and a standard deviation of 0.3:

- Negative relationships: $N(-0.3, 0.3)$
- Positive relationships: $N(0.3, 0.3)$

Strong prior

For well-documented cases, such as the negative relationship between salinity and invertebrates (e.g., [27]), an informative prior of $N(-0.2, 0.1)$ or $N(0.2, 0.1)$ was applied. This corresponds to a statistically significant relationship of $z=0.2/0.1=2$ with a p-value of 0.046. This falls within the 95% confidence interval, approximately between 0 and 0.6, for a normal distribution.

Bayesian Model Averaging (BMA)

To account for uncertainty of these different priors, we applied BMA[46,47]. This means each prior can be used incorporating all 'uncertainties' into the model. By using a Dirichlet distribution with alpha parameters of 1 for each prior odds, the prior odds are treated as equally likely, but behave stochastic rather than fixed. These assignments ensure that prior distributions align with ecological expectations, while allowing flexibility in the use of all priors (see Fig S6 below for visualization).

- Negative set: {*N*(0, 10), *N*(0, 0.5), *N*(-0.3, 0.3), *N*(-0.2, 0.1)}
- Neutral set: {*N*(0, 10), *N*(0, 0.5), *N*(-0.3, 0.3), *N*(0.3, 0.3)}
- Positive set: {*N*(0, 10), *N*(0, 0.5), *N*(0.3, 0.3), *N*(0.2, 0.1)}

Prior Assignments for Specific Stressor-Response Relationships

- Salinity increase: All freshwater organism groups (except bacteria) got a negative prior set for salinity [26,27,48]. Only the neutral prior set was used for bacteria relative to all stressors, as it was challenging to find information on this group and reach a general consensus.
- Oxygen depletion: Invertebrates and fish were assigned a negative prior set [10–12].
- Fine sediment increase: Invertebrates and fish got a negative prior set [49–51].
- Warming: Invertebrates got a negative prior set [52] and fish a positive prior set [53,54].
- Flow cessation: Macrophytes got positive prior set [55–59]and invertebrates a negative prior set [60].
- Nutrient-N and P: Only primary producers are expected to have a relation, where algae got a positive prior set [35] and macrophytes a negative prior set on richness [23,61] and a positive on coverage (%) [62]. The latter positive relation is not displayed in Supplementary Figure 6.

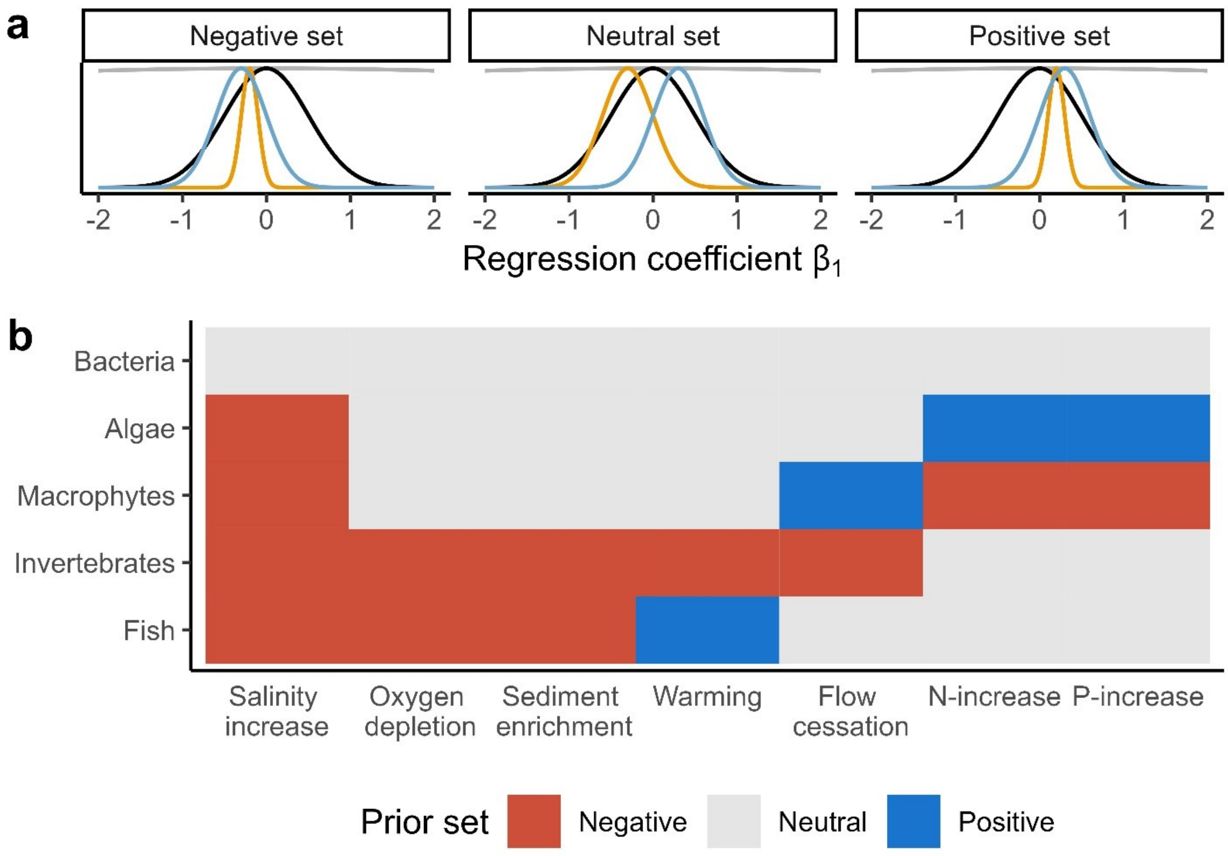

Supplementary Figure 6: Overview of prior sets used for analysis. (a) Prior sets for the regression coefficient (β_1). (b) Assignment of prior sets to stressor-response relationships.

Step 6: Meta-analysis

Each parameter estimate of the stressor-response relationship per organism group, stressor, and link function (log- and logit-linear models) was modeled separately using a random-effects meta-analysis with Bayesian Model Averaging (BMA). Since BMA employs multiple priors, the Dirichlet distribution assigns stochastic prior weights (probabilities) to each prior. Traditionally, prior odds are fixed at specific values, often set to equal weights (e.g., 1/4 = 25% for each prior). The likelihood data in the model then shifts these prior probabilities to posterior probabilities. To balance the benefits of BMA while avoiding excessive parameterization, prior weights were modeled stochastically using a Dirichlet distribution with alpha (α) weighted = 1 for each prior. The Dirichlet distribution models proportions that sum to 1, ensuring that each weight between 0 and 1 is equally likely, with the total weight summing to 1. This approach allows prior weights to vary dynamically according to the information provided by the likelihood.

Step 7: Posterior bias check

Following the meta-analysis, a posterior residual bias check was conducted by visually assessing the mean posterior residuals. Ideally, residuals should be centered at 0 and exhibit minimal to no diagonal relationship with the inverse of the standard error (1/SE), particularly with fewer missing points near 0. This relationship was visualized with a red dashed line, which should approximately align with the solid blue line, like funnel plots.

Fig. S7 illustrates that the relationship between 1/SE and the posterior estimates results in a centered distribution of residuals with no clear diagonal pattern. Additional combinations of 1/SE in relation to biotic groups (Fig. S8) and stressors (Fig. S9) were examined, with only negligible deviations observed.

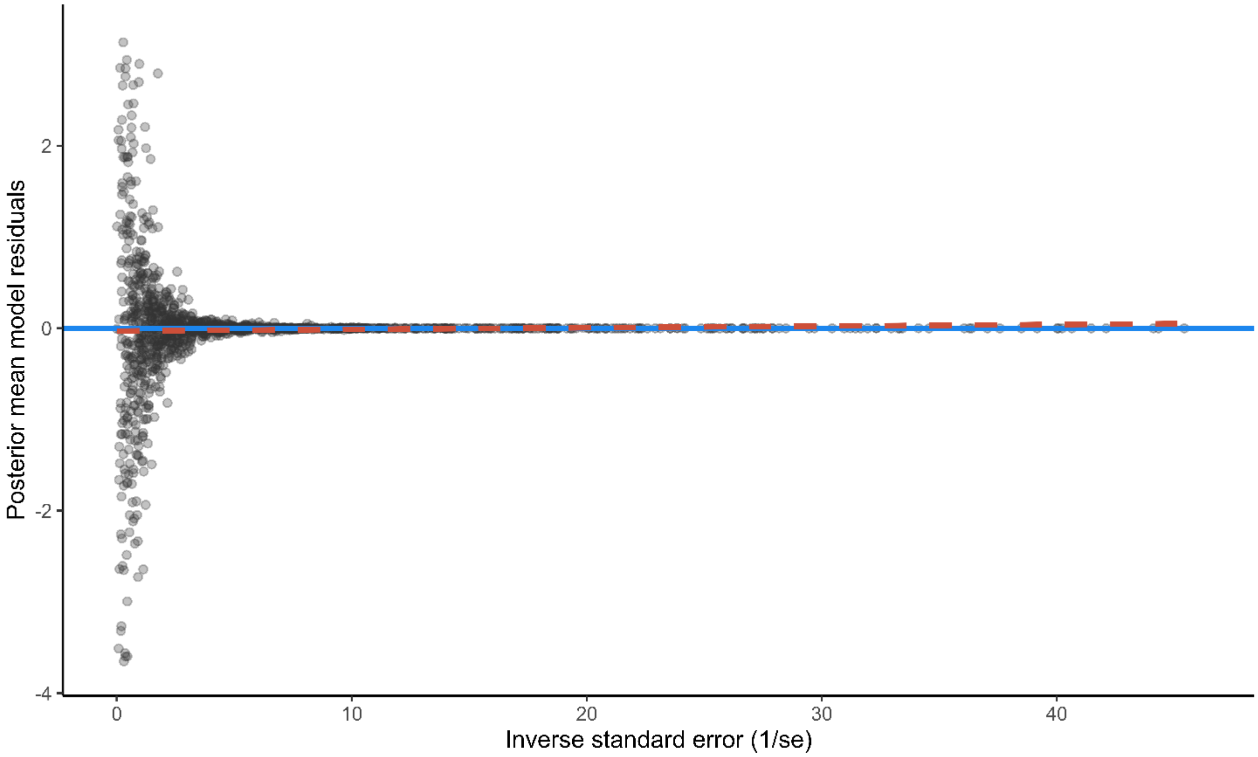

Supplementary Figure 7: Distribution of residuals as a function of 1/SE. The dashed red line should approximately align with the solid blue line.

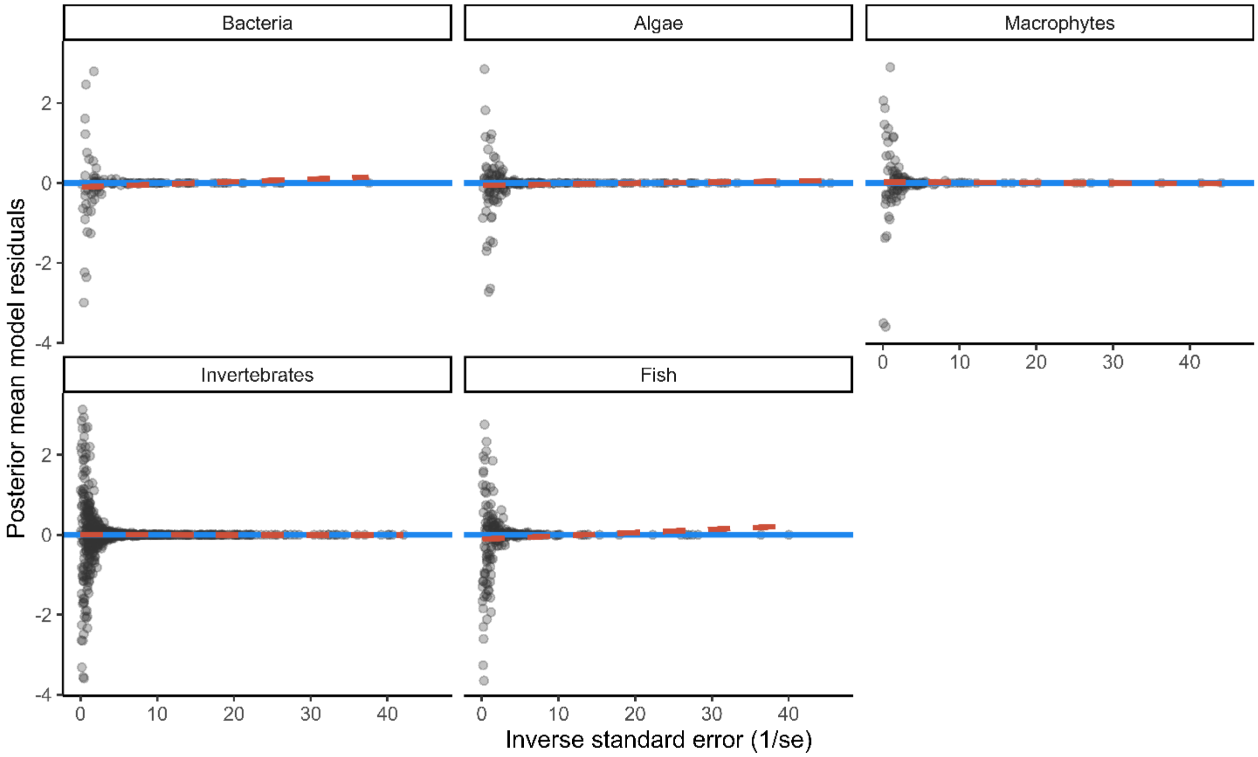

Supplementary Figure 8: Distribution of residuals for each stressor as a function of 1/SE.

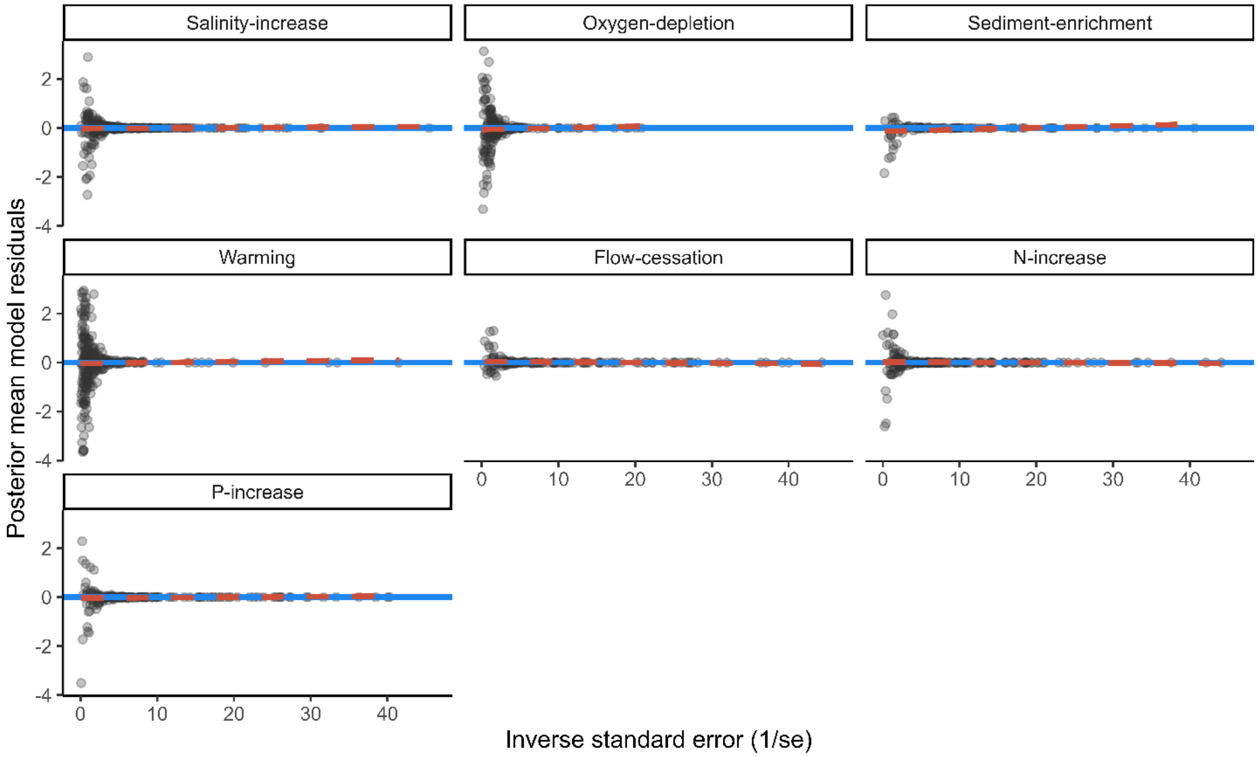

Supplementary Figure 9: Distribution of residuals for each biotic group as a function of 1/SE.

Step 8: Posterior sensitivity check

For the sensitivity analysis, the results from the main model (M1) were compared to those from a model using only a diffuse prior N(0,10) for all stressor-response relationships (M0). The natural log of the ratio between M1 and M0 was taken, yielding a log-odds ratio. This ratio quantifies how much prior models contribute to the posterior estimates. A vertical black line indicates no difference between the two models (Fig. S10).

For certain stressor-organism relations (e.g., algae and salinity, macrophytes and oxygen, sediment and bacteria, thermal stress and fish), deviations were observed. Notably, prior information often suggested more negative estimated parameters than those inferred by the data only. This trend was particularly evident in taxonomic groups with fewer observations, such as bacteria, algae, and macrophytes.

For bacteria and sediment in the logit-linear model, the log-odds ratio is negative even though a neutral prior was used. The diffuse prior as comparison for the sensitivity analysis has only minimal information. Contrary, the neutral prior contains information, but is neutral to the direction of the stressor-response relation (either N(-0.3, 0.3) or N(0.3, 0.3)). When data are sparse (n = 1 in this case) and lean toward the prior's structure, the prior exerts stronger influence. This does not reflect a directional bias, but the greater influence of prior information when data are sparse. However, as can be observed in Fig. 2 in the main text, the posterior estimate is nearly shrunk to 0. This is contrary to the only estimate in the data as -1.47 (se=0.73; Fig S2). This indicates that the likelihood information provided is limited. A similar pattern is seen for macrophytes and oxygen depletion.

For fish, the log-odds ratio is marginally positive, indicating that the main model implies a slightly less negative effect than M0.

These patterns can be consistently interpreted across other groups in Fig. S10 in combination with Fig. S2 and Fig. 2. As a general outcome, small sample sizes amplify the influence of prior assumptions on posterior estimates.

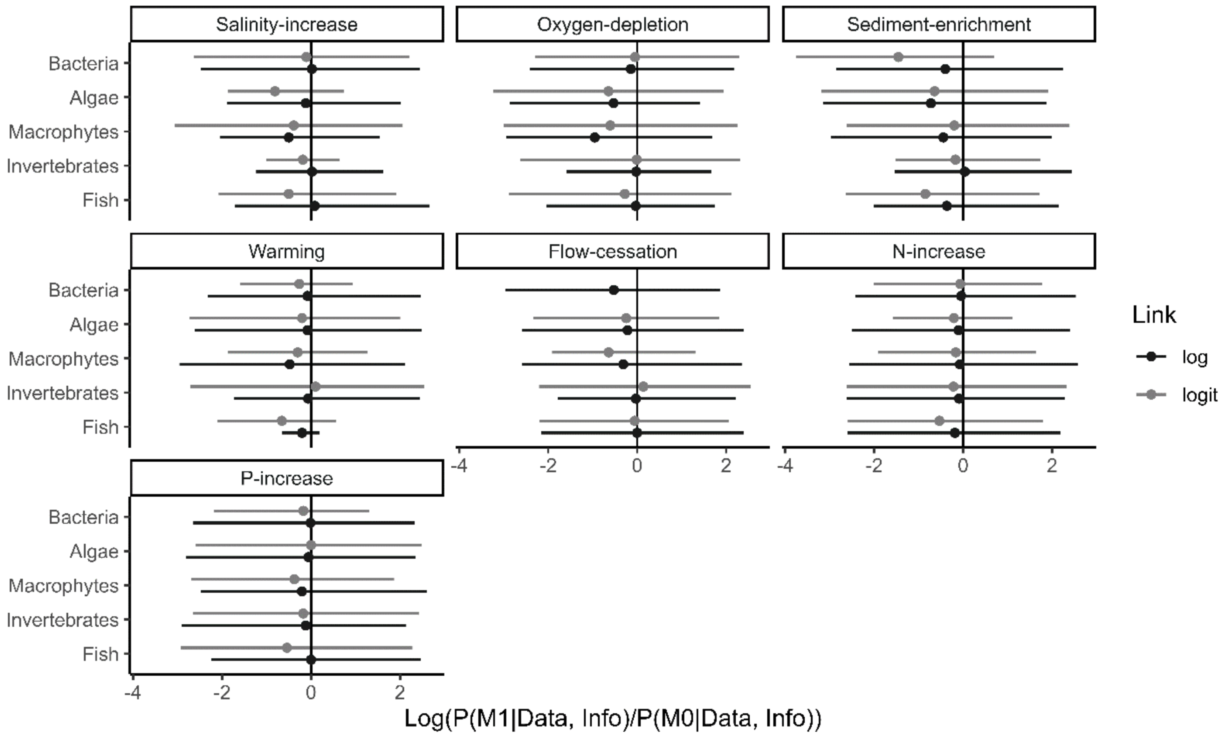

Supplementary Figure 10: Distribution of the logged odds ratio of the main model versus the uninformative diffuse model. Points represent the Maximum-A-Posteriori (MAP) estimation, while error bars indicate 90% high-density intervals. The vertical black line represents no difference between models.

It is also possible to overlay the density plots of the posterior density distribution of M1 and M0 for both log-linear (Fig. S11) and logit-linear models (Fig. S12). This shows that M0 is often more negative or positive. For the log-linear models: algae vs. oxygen-depletion, bacteria vs. sediment-enrichment, and fish vs. warming. On the other hand, some posteriors for M1 are more concentrated into a specific direction, such as Bacteria vs. flow-cessation, algae vs. warming, macrophytes vs. salinity increase, oxygen depletion, flow-cessation, and N-increase, invertebrates vs. sediment-enrichment and warming, fish vs. salinity-increase, and sediment-enrichment. The same patterns can be observed for the logit-linear models. These trends were explicitly observed for taxonomic groups with fewer observations such as bacteria and macrophyte, consistent with the previous check.

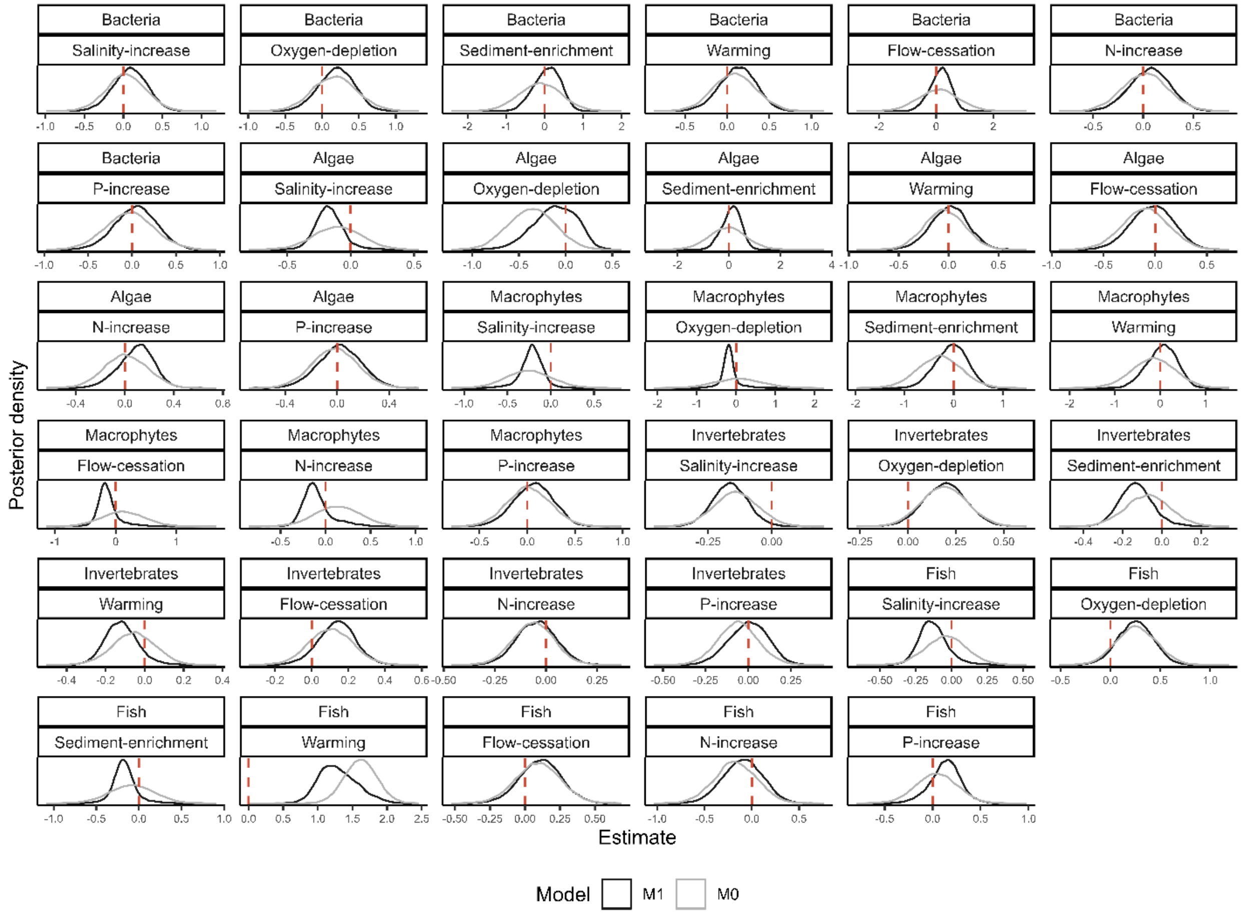

Supplementary Figure 11: Overlayed posterior density distributions for the log-linear models for M1 and M0. The red dashed line indicates 0.

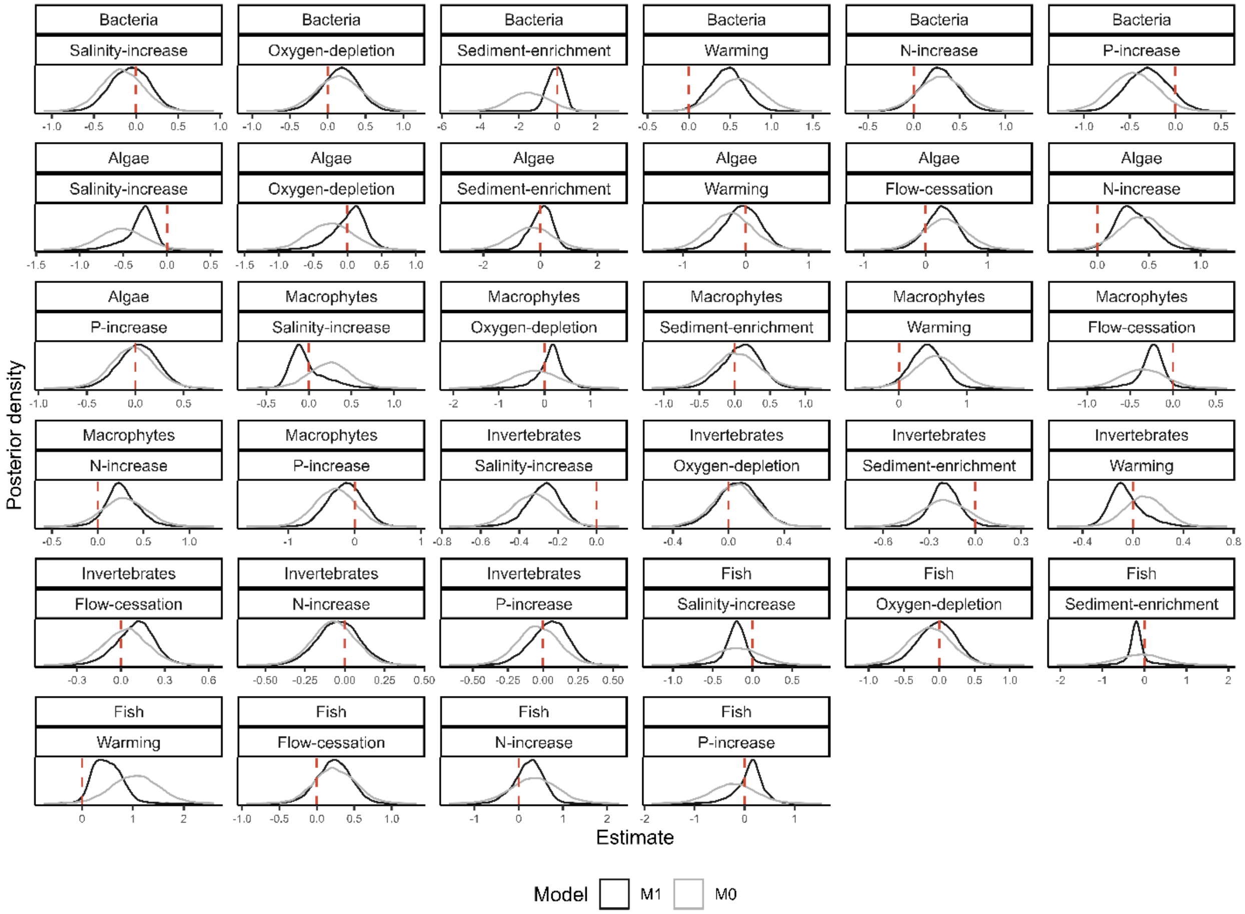

Supplementary Figure 12: Overlayed posterior density distributions for the logit-linear models for M1 and M0. The red dashed line indicates 0.

Step 9: Visualizing the results

Posterior density plots

For each pooled parameter estimate (β_0 and β_1) from the meta-analysis, the posterior distribution was obtained through Markov Chain Monte Carlo (MCMC) iterations. The results of these iterations are displayed as the posterior probability density of β_1.

Hypothetical Outcome Plots (HOPs)

Posterior estimates of $\beta_1$were used to visualize regression lines for four of the strongest relationships. The process involved:

- Generating a sequence of values within a predefined range x={x_i, …, x_n} with a fixed step size for each stressor gradient.
- Randomly sampling values from the posterior distributions of β_0 and β_1.
- Computing E(y|x) by applying the inverse link function after multiplying Log(x) with the posterior estimate.
- Repeating the process 1500 times.

The resulting lines are called Hypothetical Outcome Plots (HOPs)[63], which illustrates the expected marginal change along a stressor gradient (Eq. 1):

$$g(E(y_i|x_{ij})) = \beta_0 + \beta_j * x_{ij} + \sum_{j=1}^{k}(\beta_j * 1)\ (Eq. 1)$$

A dissimilarity with 'the classical' approach arises, given that the sum of parameter estimates is used, therefore,x_j =1 (Eq. 1). Classically, it is held constant at its mean value for each independent variable x_i (Eq. 2).

$$g(E(y_i|x_{ij})) = \beta_0 + \beta_j * x_{ij} + \sum_{j=1}^{k}(\beta_j *)\ \ (Eq. 2)$$

Partial Dependence Plots (PDPs)

PDPs convey similar information as HOPs, but with a key distinction. While HOPs depict variability by drawing samples from the posterior distribution for a single independent variable, PDPs illustrate the relationship of the expected value between two stressors. The equation is as follows:

$$g(E(y_i|x_{ij})) = \beta_0 + \beta_j * x_{ij} + \beta_k * x_{ik} + \sum_{j=1}^{k}(\beta_j * 1) \text{ (Eq. 3)}$$

PDPs are visualized as heatmaps, displaying predicted changes across a grid. However, unlike HOPs, they do not capture variability within each grid cell.

Both HOPs and PDPs serve as visual tools, rather than precise numerical predictions. The key insights lie in the curvature and spread of the plots, rather than the exact numerical values of the response.

References

1. Dolédec, S. *et al.* Multiple stressors shape invertebrate assemblages and reduce their trophic niche: A case study in a regulated stream. *Science of The Total Environment* **773**, 145061 (2021).

2. Hartig, F. Package 'DHARMa'. (2017).

3. Woolridge, J. M. *Econometric Analysis of Cross Section and Panel Data*. (The MIT press, Cambridge, Massachusetts, London, England, 2001).

4. Greenland, S., Schlesselman, J. J. & Criqui, M. H. The Fallacy of Emplying Standardized Regression Coefficients and Correlation as Measures of Effect. *American Journal of Epidemiology* **123**, 203–208 (1986).

5. Penney, J. A self-reference problem in test score normalization. *Economics of Education Review* **61**, 79–84 (2017).

6. Van Zwet, E. W. & Cator, E. A. The significance filter, the winner's curse and the need to shrink. *Statistica Neerlandica* **75**, 437–452 (2021).

7. Su, Y.-S. & Yajima, M. R2jags: Using R to Run 'JAGS'. *R package version 0.7-1* (2021).

8. Carnap, R. *Logical Foundation of Probability*. (University of Chicago Press, United States of America, 1950).

9. De Finetti, B. *Theory of Probability: A Critical Introductory Treatment*. (John Wiley & Sons, Chichester, UK Hoboken, NJ, 2017).

10. Dodds, W. K. *Freshwater Ecology: Concepts and Environmental Applications*. (Academic Press, 2002).

11. Wetzel, R. G. *Limnology: Lake and River Ecosystems*. (Academic Press, 2001).

12. Hogeboom, H. *Aquatische Ecologie*. (KNNV Uitgeverij, Zeist, 2014).

13. Champion, M. & Currie, D. J. Phosphorus—chlorophyll relationships in lakes, rivers and estuaries. *SIL Proceedings, 1922-2010* **27**, 1986–1989 (2000).

14. Chen, S., Shi, L., Shan, Z. & Hu, Q. Determination of organochlorine pesticide residues in rice and human and fish fat by simplified two-dimensional gas chromatography. *Food Chemistry* **104**, 1315–1319 (2007).

15. Dillon, P. J. & Rigler, F. H. The phosphorus-chlorophyll relationship in lakes1,2: Phosphorus-chlorophyll relationship. *Limnol. Oceanogr.* **19**, 767–773 (1974).

16. Håkanson, L. & Eklund, J. M. Relationships Between Chlorophyll, Salinity, Phosphorus, and Nitrogen in Lakes and Marine Areas. *Journal of Coastal Research* **263**, 412–423 (2010).

17. Phillips, G. *et al.* Chlorophyll–nutrient relationships of different lake types using a large European dataset. *Aquat Ecol* **42**, 213–226 (2008).

18. Pridmore, R. D., Vant, W. N. & Rutherford, J. C. Chlorophyll-nutrient relationships in North Island lakes (New Zealand). *Hydrobiologia* **121**, 181–189 (1985).

19. Seip, K. L., Jeppesen, E., Jensen, J. P. & Faafeng, B. Is trophic state or regional location the strongest determinant for Chl-a/TP relationships in lakes?: *Aquat. sci.* **62**, 195–204 (2000).

20. Stow, C. A. & Cha, Y. Are Chlorophyll *a* –Total Phosphorus Correlations Useful for Inference and Prediction? *Environ. Sci. Technol.* **47**, 3768–3773 (2013).

21. Wagner, T., Soranno, P. A., Webster, K. E. & Cheruvelil, K. S. Landscape drivers of regional variation in the relationship between total phosphorus and chlorophyll in lakes: Relationship between total phosphorus and chlorophyll. *Freshwater Biology* **56**, 1811–1824 (2011).

22. Lorenz, A. W. *et al.* Stressors affecting the ecological status of temporary rivers in the Mediterranean region. *Science of The Total Environment* **903**, 166254 (2023).

23. Kaijser, W., Hering, D. & Kail, J. Macrophyte growth forms shift along the trophic gradient of lakes. *Inland Waters* **13**, 402–411 (2024).

24. Kaijser, W. *et al.* Differential associations of five riverine organism groups with multiple stressors. *Science of The Total Environment* **934**, 173105 (2024).

25. Greenop, K. R., Stewart, B. A. & Close, P. G. Can a naturally depauperate Ephemeroptera, Plectoptera and Trichoptera (EPT) fauna track river degradation in south-western Australia? *Environ Monit Assess* **196**, 592 (2024).

26. Moors, W. *et al. Levensgemeenschappen Van Brakke Wateren [Eng: Communities of Brackish Waters]*. 13 (1995).

27. Remane, A. & Schlieper, C. *The Biology of Brackish Waters*. (E. Schweizerbart'sche Verlag, Stuttgart, 1972).

28. Welti, E. A. R. *et al.* Time series of freshwater macroinvertebrate abundances and site characteristics of European streams and rivers. *Sci Data* **11**, 601 (2024).

29. Bistoni, M. A. & Hued, A. C. Patterns of fish species richness in rivers of the central region of Argentina. *Braz. J. Biol.* **62**, 753–764 (2002).

30. Eckmann, R. Fish species richness in lakes of the northeastern lowlands in Germany. *Ecology of Freshwater Fish* **4**, 62–69 (1995).

31. Kaijser, W., Birk, S. & Hering, D. Environmental ranges discriminating between macrophytes groups in European rivers. *PLoS ONE* **17**, e0269744 (2022).

32. Kaijser, W., Hering, D. & Lorenz, A. W. Reach hydromorphology: a crucial environmental variable for the occurrence of riverine macrophytes. *Hydrobiologia* **849**, 4273–4285 (2022).

33. Masese, F. O. *et al.* Bioassessment of multiple stressors in Afrotropical rivers: Evaluating the performance of a macroinvertebrate-based index of biotic integrity, diversity, and regional biotic indices. *Front. Environ. Sci.* **11**, 1015623 (2023).

34. Pusey, B. J., Arthington, A. H. & Read, M. G. Species richness and spatial variation in fish assemblage structure in two rivers of the Wet Tropics of northern Queensland, Australia. *Environ Biol Fish* **42**, 181–199 (1995).

35. Stomp, M., Huisman, J., Mittelbach, G. G., Litchman, E. & Klausmeier, C. A. Large-scale biodiversity patterns in freshwater phytoplankton. *Ecology* **92**, 2096–2107 (2011).

36. Stouten, P. J. *Verslag Hydrobiologische Waterkwaliteitsbepaling 1990 en 1991 van Waterschap Hulster Ambacht [Eng: Report Hydrobiological Water Quality Assessment 1990 and 1991 of Hulster Ambacht Water Board]*. 1–12 (1992).

37. Súarez, Y. R. *et al.* Patterns of species richness and composition of fish assemblages in streams of the Ivinhema River basin, Upper Paraná River. *Acta Limnol. Bras.* **23**, 177–188 (2011).

38. Theroux, S. *et al.* Predictive biological indices for algae populations in diverse stream environments. *Ecological Indicators* **119**, 106421 (2020).

39. Walters, K. E. & Martiny, J. B. H. Alpha-, beta-, and gamma-diversity of bacteria varies across habitats. *PLoS ONE* **15**, e0233872 (2020).

40. Zbinden, Z. D., Geheber, A. D., Lehrter, R. J. & Matthews, W. J. Multifaceted assessment of stream fish alpha and beta diversity using spatial models. *Hydrobiologia* **849**, 1795–1820 (2022).

41. Jackson, M. C., Loewen, C. J. G., Vinebrooke, R. D. & Chimimba, C. T. Net effects of multiple stressors in freshwater ecosystems: a meta-analysis. *Global Change Biology* **22**, 180–189 (2016).

42. Ruaro, R., Gubiani, É. A., Hughes, R. M. & Mormul, R. P. Global trends and challenges in multimetric indices of biological condition. *Ecological Indicators* **110**, 105862 (2020).

43. Sabater, S. *et al.* Effects of human-driven water stress on river ecosystems: a meta-analysis. *Sci Rep* **8**, 11462 (2018).

44. Hering, D. *et al.* Assessment of European streams with diatoms, macrophytes, macroinvertebrates and fish: a comparative metric-based analysis of organism response to stress. *Freshwater Biology* **51**, 1757–1785 (2006).

45. Mack, L. *et al.* Perceived multiple stressor effects depend on sample size and stressor gradient length. *Water Research* **226**, 119260 (2022).

46. Hinne, M., Gronau, Q. F., Van Den Bergh, D. & Wagenmakers, E.-J. A Conceptual Introduction to Bayesian Model Averaging. *Advances in Methods and Practices in Psychological Science* **3**, 200–215 (2020).

47. Hoeting, J. A., Madigan, D., Raftery, A. E. & Volinsky, C. T. Bayesian Model Averaging: A Tutorial. *Statistical Science* **14**, 382–417 (1999).

48. Bloemendaal, F. H. J. L. & Roelofs, J. G. M. *Waterplanten En Waterkwaliteit 'Netherlands' [Eng: Water Plants and Water Quality]*. (Stichting Uitgeverij van de Koninklijke Natuurhistorische Vereniging, Utrecht, 1988).

49. Kukuła, K. & Bylak, A. Synergistic impacts of sediment generation and hydrotechnical structures related to forestry on stream fish communities. *Science of The Total Environment* **737**, 139751 (2020).

50. McKenzie, M. *et al.* Freshwater invertebrate responses to fine sediment stress: A multi-continent perspective. *Global Change Biology* **30**, e17084 (2024).

51. Stoffers, T. *et al.* Freshwater fish biodiversity restoration in floodplain rivers requires connectivity and habitat heterogeneity at multiple spatial scales. *Science of The Total Environment* **838**, 156509 (2022).

52. Croijmans, L., De Jong, J. F. & Prins, H. H. T. Oxygen is a better predictor of macroinvertebrate richness than temperature—a systematic review. *Environ. Res. Lett.* **16**, 023002 (2021).

53. Guégan, J.-F., Lek, S. & Oberdorff, T. Energy availability and habitat heterogeneity predict global riverine fish diversity. *Nature* **391**, 382–384 (1998).

54. Xia, Z. *et al.* Elevational patterns of fish functional and phylogenetic community structure in a monsoon climate river basin. *Diversity and Distributions* **30**, e13815 (2024).

55. Biggs, B. J. F. Hydraulic habitat of plants in streams. *Regulated rivers: Research & Managment* **12**, 131–144 (1996).

56. Haslam, S. M. *River Plants of Western Europe*. (Cambridge University Press, Cambridge, United Kingdom, 1987).

57. Haslam, S. M. *River Plants: The Macrophytic Vegetation of Watercourses*. (Cambridge University Press, Cambridge, 1978).

58. Janauer, G. A., Gaberščik, A., Květ, J., Germ, M. & Exler, N. *Macrophytes of the River Danube Basin*. (Academia, 2018).

59. Riis, T. & Biggs, B. J. F. Hydrologic and hydraulic control of macrophyte establishment and performance in streams. *Limnol. Oceanogr.* **48**, 1488–1497 (2003).

60. Bartels, A., Berninger, U. G., Hohenberger, F., Wickham, S. & Petermann, J. S. Littoral macroinvertebrate communities of alpine lakes along an elevational gradient (Hohe Tauern National Park, Austria). *PLoS ONE* **16**, e0255619 (2021).

61. Poikane, S., Várbíró, G., Kelly, M. G., Birk, S. & Phillips, G. Estimating river nutrient concentrations consistent with good ecological condition: More stringent nutrient thresholds needed. *Ecological Indicators* **121**, 107017 (2021).

62. Verhofstad, M. J. J. M. *et al.* Mass development of monospecific submerged macrophyte vegetation after the restoration of shallow lakes: Roles of light, sediment nutrient levels, and propagule density. *Aquatic Botany* **141**, 29–38 (2017).

63. Kale, A., Nguyen, F., Kay, M. & Hullman, J. Hypothetical Outcome Plots Help Untrained Observers Judge Trends in Ambiguous Data. *IEEE Trans. Visual. Comput. Graphics* **25**, 892–902 (2019).
